# Thermal Compatibility of New ACEI Derivatives with Popular Excipients Used to Produce Solid Pharmaceutical Formulations

**DOI:** 10.3390/ph17101323

**Published:** 2024-10-03

**Authors:** Mateusz Broncel, Anna Juszczak, Wojciech Szczolko, Daniele Silvestri, Agnieszka Białek-Dratwa, Stanisław Wacławek, Oskar Kowalski, Paweł Ramos

**Affiliations:** 1 Department of Biophysics, Faculty of Pharmaceutical Sciences in Sosnowiec, Medical University of Silesia, Jedności 8, 41-200 Sosnowiec, Poland; 2 Doctoral School, Medical University of Silesia, Jedności 8, 41-200 Sosnowiec, Poland; 3 Chair and Department of Pharmaceutical Chemistry, Poznan University of Medical Sciences, Rokietnicka 3, 60-806 Poznan, Poland; anna.juszczak28@gmail.com; 4 Chair and Department of Chemical Technology of Drug, Poznan University of Medical Sciences, Rokietnicka 3, 60-806 Poznan, Poland; wszczolko@ump.edu.pl; 5 Institute for Nanomaterials, Advanced Technologies and Innovation, Technical University of Liberec, Stdentská 2, 460 01 Liberec, Czech Republic; daniele.silvestri@tul.cz (D.S.); stanislaw.waclawek@tul.cz (S.W.); 6 Department of Human Nutrition, Department of Dietetics, Faculty of Public Health in Bytom, Medical University of Silesia, Jordana 19, 41-808 Zabrze, Poland; abialek@sum.edu.pl (A.B.-D.); okowalski@sum.edu.pl (O.K.)

**Keywords:** ACEI, pharmaceutical excipients, compatibility, TGA, c-DTA, FTIR, colorimetry analysis, UV spectroscopy

## Abstract

**Background/Objectives:** Increasing drugs’ stability and adequately protecting them against degradation will ensure a decrease in their price and broader availability of pharmaceutical substances. This is of great importance, especially for drugs used to treat the most common diseases in the population, such as hypertension. The study examined two newly synthesized substances from the angiotensin I-converting enzyme inhibitor (ACEI) group as potential drugs. ACEIs are among the leading drugs used in the treatment of hypertension in the world. The chemical modifications of the tested substances applied concerned the places most susceptible to degradation. The presented work analyzed the compatibility of new derivatives with selected excipients used in pharmacy. **Methods**: Thermogravimetric (TGA) and differential thermal analyses (c-DTA) were used as the main methods. In addition, non-thermal methods such as colorimetry analysis, Fourier-transform infrared (FTIR) and UV spectroscopy were used. Results: Based on the conducted studies, it can be concluded that the incompatibility of IND-1 with glucose anhydrous and lactose monohydrate occurs only when the mixture is stored at higher temperatures. For the remaining IND-1 and IND-2 mixtures with excipients, compatibility was demonstrated. **Conclusions**: The obtained results confirmed the usefulness of the applied thermal analyses (TGA and c-DTA) for assessing the compatibility of the tested potential drugs with excipients. However, in the case of incompatibility reactions of substances occurring under the influence of elevated temperatures, such as the Maillard reaction, it is necessary to use non-thermal methods to obtain the right result.

## 1. Introduction

The rapid development of civilization directly contributes to the increase in the incidence of lifestyle diseases, which include, among others, hypertension and ischemic heart diseases. This is a challenge for modern pharmacy and sets the direction for the search for new drugs that will allow for effective and cheap treatment of diseases whose incidence is increasing every year. Increasing drugs’ stability and properly protecting them against degradation will ensure a decrease in their price and wider availability of pharmaceutical substances [1]. 

Angiotensin I-converting enzyme inhibitors (ACEIs) are among the leading drugs used in the treatment of hypertension in the world. More indications for their use are still being discovered [2,3,4,5,6]. ACEIs owe their versatile use to their complex mechanism of action. The general mechanism of action of ACEIs is to reduce the formation of angiotensin II, which lowers blood pressure [2]. In addition to the hypotensive effect, ACEIs have antiproliferative, nephroprotective and even antithrombotic effects [3,4]. The antioxidant effect of popular ACEI drugs has also been demonstrated [5,6]. ACEIs are used in monotherapy for mild to moderate hypertension and in more severe forms in combination therapy. Positive features of most compounds from this group are a mild onset of action and a long-lasting hypotensive effect, and the use of the preparations in one daily dose facilitates systematic long-term pharmacotherapy [2,7]. 

However, despite the many advantages of ACEIs, numerous studies have shown the low stability of these drugs at elevated temperatures and relative air humidity [8,9,10,11]. This is particularly important during the long-term storage of these drugs. Therefore, despite the wide range of this therapeutic group, new compounds with the same pharmacological profile but with increased durability are constantly being searched for.

The final medicinal product comprises an active pharmaceutical substance (API) and an excipient. Excipients are intended to give a drug the appropriate shape and mass, improving stability and control release and providing lubricating properties to the final form of the drug [1,12,13]. However, excipients cannot react with the API; such a phenomenon could have an adverse effect on the drug’s action, its release and metabolism, which may result in undesirable effects that are difficult to predict [1,14]. Therefore, it is essential to perform analyses to check whether a given excipient does not interact with the drug’s active pharmaceutical ingredient. 

The study examined two newly synthesized substances as potential drugs from the ACEI group. The synthesis was carried out to create derivatives with more excellent stability. The chemical modifications of the tested substances applied concerned the places most susceptible to degradation. The presented study analyzed the thermal decomposition profile and compatibility of new derivatives, such as 2-(benzylamine)-1-(2,3-dihydro-1H-indol-1-yl) ethenone (IND-1) and 2-chloro-1-(2, 3-dihydro-1H-indol-1-yl) propoan-1-one (IND-2), with selected excipients used in pharmacy. Among the excipients selected for the study were glucose monohydrate, lactose monohydrate, microcrystalline cellulose, starch from corn and magnesium stearate. These substances were selected because of their wide use in solid-dosage-form formulations. Most of them are used in commercially available pharmaceutical preparations containing ACEIs. In order to assess the compatibility of the tested derivatives with selected excipients, thermogravimetric (TGA) and differential thermal analyses (c-DTA) were used. Moreover, to evaluate interactions, colorimetry analysis, Fourier-transform infrared (FTIR) and UV spectroscopy were used. The chemical structure of the tested derivatives was confirmed using nuclear magnetic resonance spectroscopy (NMR).

## 2. Results and Discussion

### 2.1. Synthesis and Confirmation of the Chemical Structure of the New ACEI Derivatives Tested

Known compounds **1** [15] and **2** [16] were synthesized according to the procedure described in the literature with modifications. 

**Figure 1:**
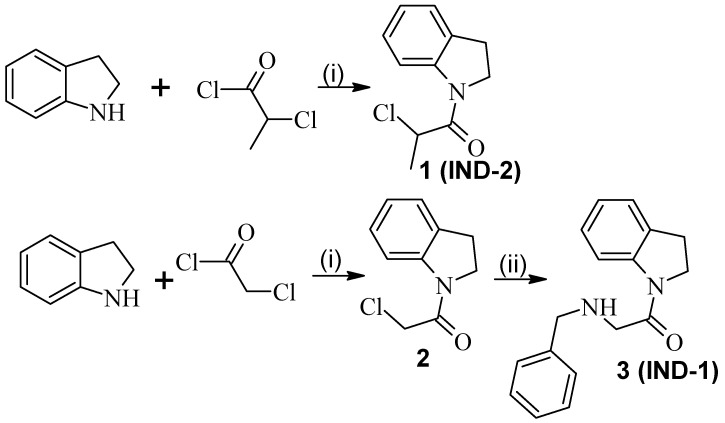
Conditions of reaction: (i) THF, 30 min room temperature; (ii) benzylamine, K_2_CO_3_, KI, re-flux, 24 h.

Indoline was used in an acylation reaction with chloroacetyl chloride and 2-chloropropionyl chloride in THF to give, respectively, derivative **1** (IND-2) and 2. Next, derivative 1 was used in an alkylation reaction with benzylamine in THF with potassium carbonate and potassium iodide to give derivative **3** (IND-1) (Figure 1). 

All compounds were characterized by MS ES, UV–Vis, and ^1^H and ^13^C NMR (Supplementary Materials). 

### 2.2. Assessment of the Compatibility of IND-1 and IND-2 Derivatives with Selected Excipients Using the Thermal Methods

Thermal analyses such as thermogravimetry (TG) and differential thermal analysis (c-DTA) were used in the research. Thermal methods were used to detect interactions between newly synthesized ACEI derivatives and excipients commonly used in the pharmaceutical industry. Among the commonly used excipients in the work, glucose anhydrous, lactose monohydrate, microcrystalline cellulose, starch from corn and magnesium stearate were used. In pharmacy, glucose anhydrous is used as a diluent for tablets and capsules and tonic agents. Lactose monohydrate is widely used in the pharmaceutical industry as a tablet binder, a tablet and capsule diluent, and a tablet and capsule filler, among other uses. Microcrystalline cellulose may be used as a tablet and capsule diluent, disintegrant, adsorbent and suspending agent. Starch from corn may be used as a tablet and capsule filler, binding agent, compression aid, disintegrant, tablet and capsule diluent. In pharmacy, chitosan may be used as a coating agent, tablet disintegrant, tablet binder, viscosity-increasing agent and mucoadhesive. Magnesium stearate is used as a lubricant in tablets and capsules [17].

As previous studies have shown, thermal methods can be used to initially assess the compatibility of APIs with excipients [18,19,20]. TGA, DTA and DSC are eagerly used to assess non-compliance because they have many advantages. First, they require a small volume of the test sample, are quick to perform and are inexpensive.

**Figure 2:**
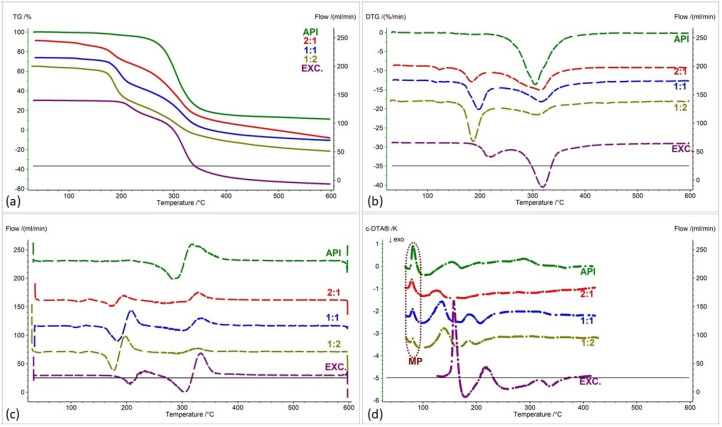
(**a**) TG, (**b**) DTG, (**c**) D2TG and (**d**) c-DTA curves of IND-1 (API), glucose anhydrous (EXC.) and binary mixture of API and excipient in different weight ratios (2:1, 1:1 and 1:2). MP—melting point.

**Figure 3:**
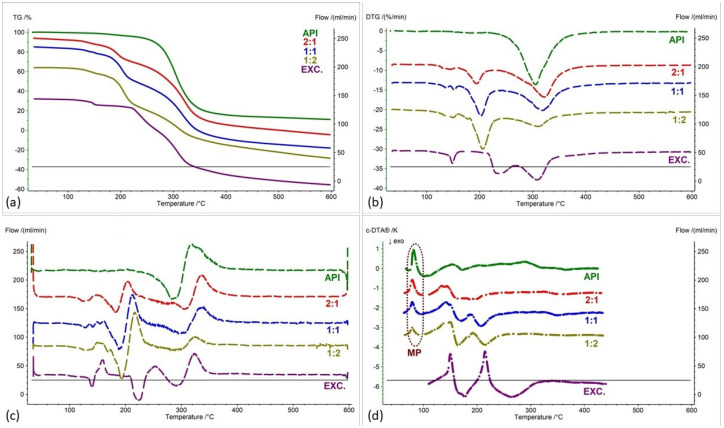
(**a**) TG, (**b**) DTG, (**c**) D2TG and (**d**) c-DTA curves of IND-1 (API), lactose monohydrate (EXC.) and binary mixture of API and excipient in different weight ratios (2:1, 1:1 and 1:2). MP—melting point.

**Figure 4:**
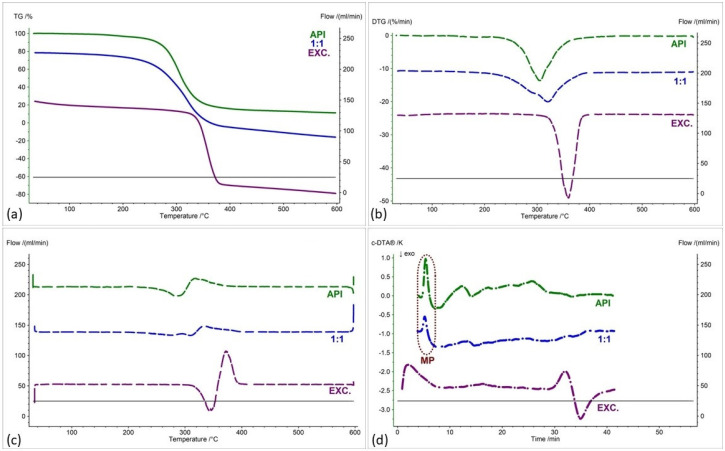
(**a**) TG, (**b**) DTG, (**c**) D2TG and (**d**) c-DTA curves of IND-1 (API), microcrystalline cellulose (EXC.) and binary mixture of API and excipient in a weight ratio of 1:1. MP—melting point.

**Figure 5:**
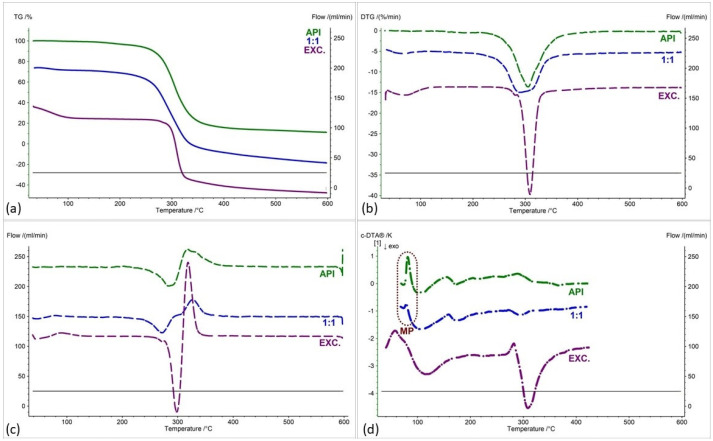
(**a**) TG, (**b**) DTG, (**c**) D2TG and (**d**) c-DTA curves of IND-1 (API), starch from corn (EXC.) and binary mixture of API and excipient in a weight ratio of 1:1. MP—melting point.

**Figure 6:**
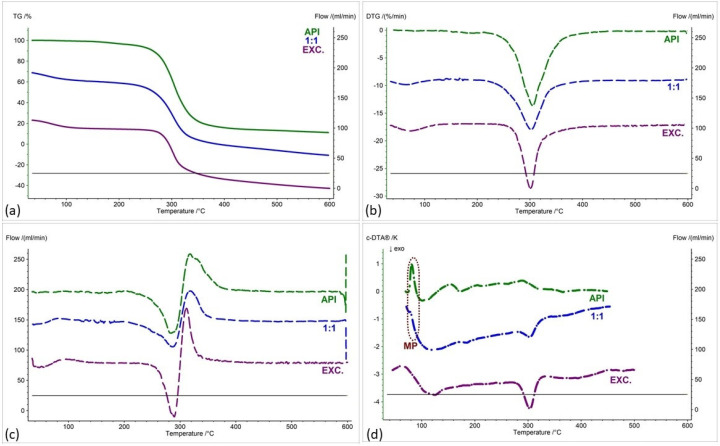
(**a**) TG, (**b**) DTG, (**c**) D2TG and (**d**) c-DTA curves of IND-1 (API), chitosan (EXC.) and binary mixture of API and excipient in a weight ratio of 1:1. MP—melting point.

**Figure 7:**
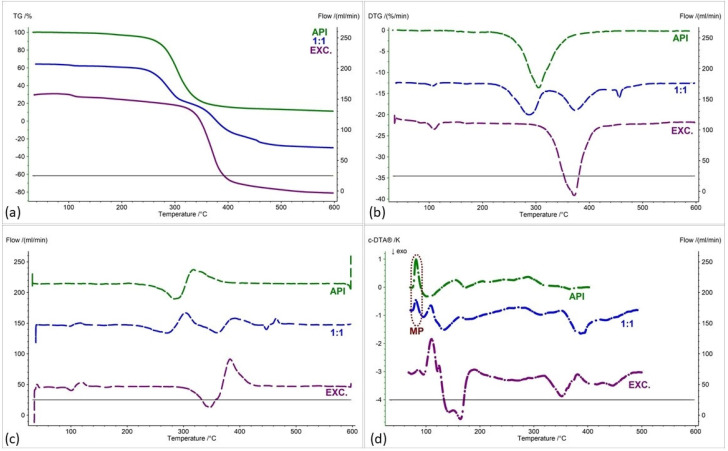
(**a**) TG, (**b**) DTG, (**c**) D2TG and (**d**) c-DTA curves of IND-1 (API), magnesium stearate (EXC.) and binary mixture of API and excipient in a weight ratio of 1:1. MP—melting point.

Figure 2, Figure 3, Figure 4, Figure 5, Figure 6 and Figure 7 show thermogravimetric curves (a), the first derivative of the TG curve (DTG) (b), the second derivative of the TG curve (D2TG) (c) and c-DTA curves (d) for the IND-1 compound (green curves), pure excipients (purple curves) and API mixtures with excipients.

The TG curve of the IND-1 derivative shows a one-step thermal decomposition (Figure 2a, Figure 3a, Figure 4a, Figure 5a, Figure 6a and Figure 7a, green curves). The beginning of thermal decomposition starts at a temperature of 275.7 °C. The single-stage course of decomposition was also confirmed by the DTG (Figure 2b, Figure 3b, Figure 4b, Figure 5b, Figure 6b and Figure 7b, green curves) and D2TG (Figure 2c, Figure 3c, Figure 4c, Figure 5c, Figure 6c and Figure 7c, green curves) curves. The maximum mass loss recorded on the DTG curve took place at a temperature of 305.4 °C and was 13%/min. The melting point recorded on the c-DTA curve for IND-1 is 81.5 °C (Figure 2d, Figure 3d, Figure 4d, Figure 5d, Figure 6d and Figure 7d, green curves). An endothermic peak in the c-DTA curve was recorded for the melting point. The recorded peak has a narrow, sharp shape, which indicates the high purity of the obtained derivative [21,22]. 

Thermograms obtained for pure excipients have a typical course described in the literature [23,24,25,26,27,28,29,30,31,32,33,34,35,36,37]. All thermograms obtained for individual excipients are shown in purple in the figures.

In order to assess possible interactions between the tested derivatives and excipients, mixtures of single APIs with single excipients were made in a weight ratio of 1 to 1. In the next stage, the thermograms obtained for the mixtures were compared with the thermograms recorded for the pure derivatives. Any changes in the curve parameters indicate possible interactions between the tested API and the excipient [18,19,28,36,37,38,39]. In case of an inconclusive result, mixtures of the API with the excipient were made in other weight ratios (2:1 and 1:2). If there is no interaction, as the amount of excipient in the mixture increases, the thermogram should resemble the curve recorded for the pure excipient [18]. Additional mixtures were made for IND-1 in combination with glucose anhydrous (Figure 3) and lactose monohydrate (Figure 4). In the case of IND-2, they were made in combination with starch from corn (Figure 11).

**Figure 8:**
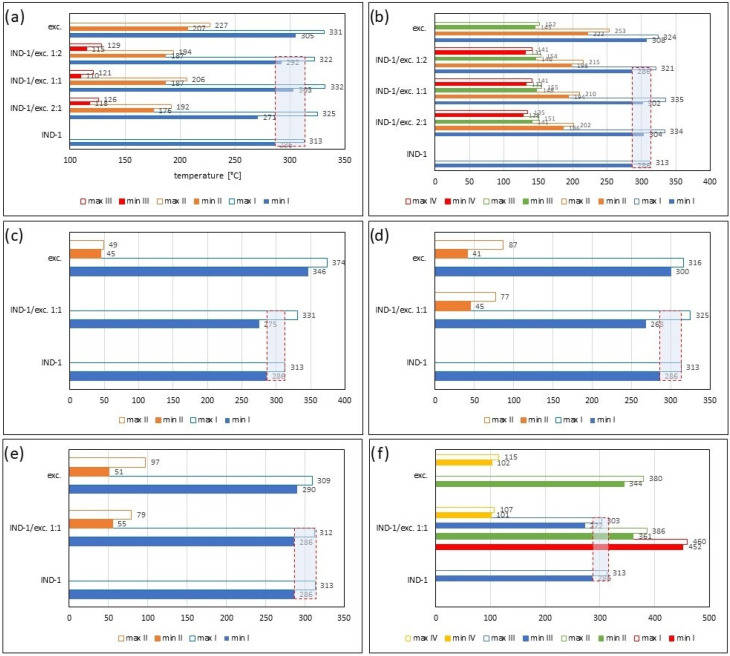
Temperature ranges recorded on D2TG curves for IND-1 (API): (**a**) glucose anhydrous, (**b**) lactose monohydrate, (**c**) microcrystalline cellulose, (**d**) starch from corn, (**e**) chitosan and (**f**) magnesium stearate; binary mixture of API and excipients in weight ratios of (**a**–**f**) 1:1 and (**a**,**b**) 2:1 and 1:2. **Min**—initial temperature peak recorded on the D2TG curve; **Max**—final temperature peak recorded on the DTG curve; **I**, **II**, **III**, **IV**—subsequent stages of decomposition. The blue boxes with red frames show the temperature ranges of the decomposition stage recorded for IND-1 and its location in the decomposition steps of the IND-1–excipient mixtures.

Figure 2 and Table 1 and Table 2 present the recorded results for IND-1, anhydrous glucose and mixtures of both substances in weight ratios of 2:1, 1:1 and 1:2. On the TG curve, we can observe a shift of the beginning of decomposition by 101.2 °C (ratio 1:2) and 28.5 °C (ratio 2:1) towards a temperature lower than for the pure drug. This may indicate an interaction between the two substances. Moreover, by analyzing the DTG curve (Figure 2b, Table 2) and D2TG curves (Figure 2c and Figure 8a), we can observe the formation of an additional peak in the temperature range of 115 °C–120 °C for the mixture in the ratio of 1:2, in the range of 110 °C–120 °C for the mixture in the ratio of 1:1 and in the range of 118 °C–126 °C for the mixture in the ratio of 2:1. This peak is not visible for the DTG and D2TG curves recorded for pure IND-1 and pure anhydrous glucose. The appearance of additional peaks in thermograms for the API–excipient mixture may indicate incompatibility [18,28,36,37,38,39].

Figure 3 as well as Table 1 and Table 2 present the thermogravimetric results recorded for the IND-1 derivative, lactose monohydrate and IND-1 with the lactose mixture. Similarly to the case of glucose anhydrous (Figure 2, Table 1), in the TG curve of the mixture of IND-1 with lactose monohydrate, we observed a shift in the onset of the decomposition temperature towards a lower temperature compared to pure IND-1 (Figure 3, Table 1). This shift was more significant when the amount of excipient in the mixture increased (samples prepared in ratios of 1:1 and 1:2). This may indicate that IND-1 is incompatible with lactose monohydrate. 

However, the analysis of c-DTA curves recorded for mixtures of IND-1 with glucose anhydrous (Figure 2d, Table 3) and lactose monohydrate (Figure 3d, Table 3) showed no change in the melting point compared to pure IND-1. This may indicate an apparent incompatibility that occurs only at higher temperatures, i.e., when the finished pharmaceutical product is improperly stored. The Maillard reaction can be responsible for these interactions [14,18]. The Maillard reaction occurs between a reducing sugar and a drug’s primary or secondary amino group [14,18,28]. The Maillard reaction occurs more quickly at higher temperatures. It is particularly dangerous when drugs are stored incorrectly. It can cause the active pharmaceutical ingredient to decompose and the finished drug formulation to change color [14,18].

To confirm the incompatibility IND-1 with glucose anhydrous and lactose monohydrate, colorimetric and FTIR measurements were additionally performed and presented in Section 2.3 and Section 2.4 of this work.

In the case of other mixtures of IND-1 with microcrystalline cellulose (Figure 4 and Figure 8, Table 1 and Table 2), starch from corn (Figure 5 and Figure 8, Table 1 and Table 2), chitosan (Figure 6 and Figure 8, Table 1 and Table 2) and magnesium stearate (Figure 7 and Figure 8, Table 1 and Table 2), no clear shift in the onset of the decomposition temperature was observed compared to pure IND-1, which indicates the compatibility of these mixtures. The recorded thermogravimetric curves for the tested mixtures show the course of pure IND-1 (Figure 4a–c, Figure 5a–c and Figure 6a–c) or contain clearly separated thermal events originating from IND-1 and the excipient (Figure 7a–c). The recorded c-DTA curves also confirm the compatibility of these mixtures (Figure 4d, Figure 5d, Figure 6d and Figure 7d, Table 3). We did not observe any shift in the melting point of the API used in the mix.

**Figure 9:**
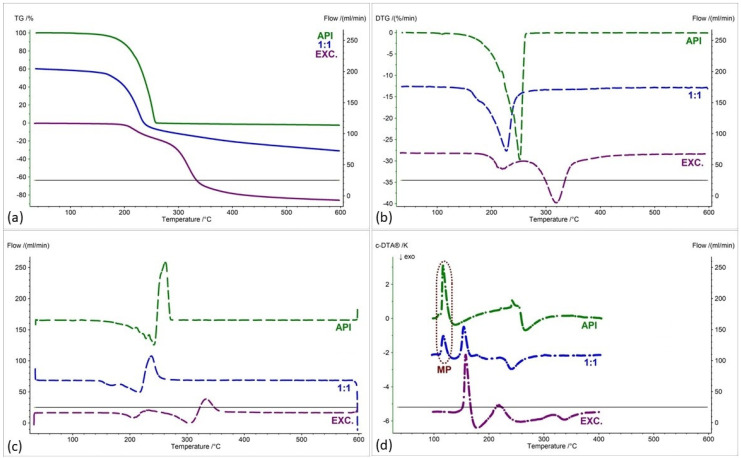
(**a**) TG, (**b**) DTG, (**c**) D2TG and (**d**) c-DTA curves of IND-2 (API), glucose anhydrous (EXC.) and binary mixture of API and excipient in a weight ratio of 1:1. MP—melting point.

**Figure 10:**
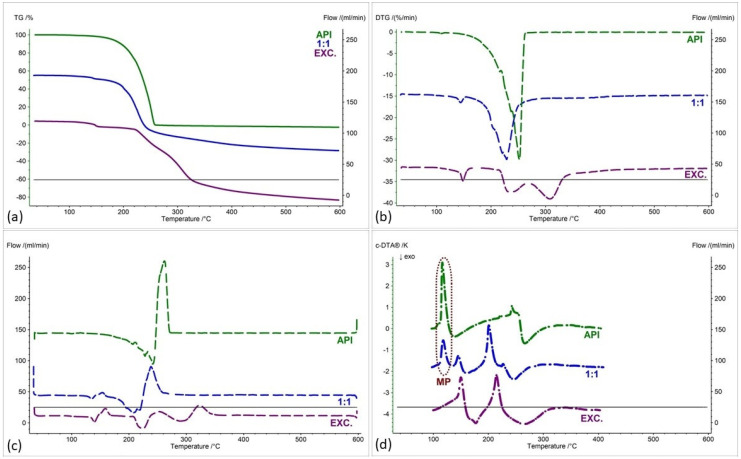
(**a**) TG, (**b**) DTG, (**c**) D2TG and (**d**) c-DTA curves of IND-2 (API), lactose monohydrate (EXC.) and binary mixture of API and excipient in a weight ratio of 1:1. MP—melting point.

**Figure 11:**
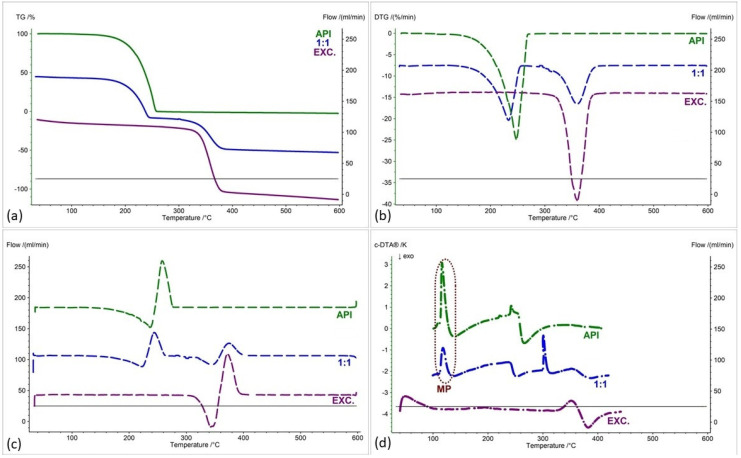
(**a**) TG, (**b**) DTG, (**c**) D2TG and (**d**) c-DTA curves of IND-2 (API), microcrystalline cellulose (EXC.) and binary mixture of API and excipient in a weight ratio of 1:1. MP—melting point.

**Figure 12:**
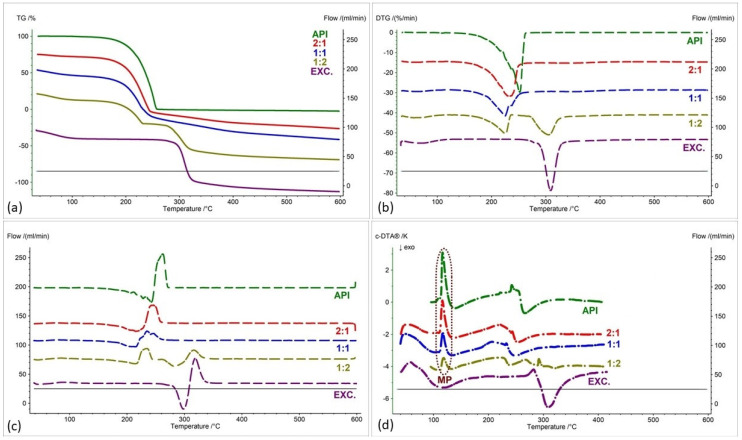
(**a**) TG, (**b**) DTG, (**c**) D2TG and (**d**) c-DTA curves of IND-2 (API), starch from corn (EXC.) and binary mixture of API and excipient in different weight ratios (2:1, 1:1 and 1:2). MP—melting point.

As with the IND-1 derivative, the IND-2 derivative is summarized in Figure 9, Figure 10, Figure 11 and Figure 12, showing (a) thermogravimetric curves, (b) the first derivative of the TG curve (DTG), (c) the second derivative of the TG curve (D2TG) and (d) c-DTA curves. As with the IND-1 derivative, the IND-2 derivative is shown in the figures as green curves and the pure excipients are shown as purple curves. In turn, mixtures of IND-2 with excipients are marked with blue curves, and in the case of starch from corn, they also have red and yellow curves.

The thermogravimetry curve of the IND-2 derivative, like the IND-1 derivative, shows a one-step thermal decomposition (Figure 9a, Figure 10a, Figure 11a and Figure 12a, green curves; Table 4). Compared to IND-1, the IND-2 derivative is less thermally stable and undergoes thermal decomposition at a temperature of 224.7 °C. The maximum mass loss rate for the IND-2 sample recorded on the DTG curve occurs at a temperature of 250 °C (29.72%/min) (Figure 9b, Figure 10b, Figure 11b and Figure 12b, green curves; Table 5). The melting point recorded on the c-DTA curve for IND-2 is higher than for the IND-1 compound and is 116.4 °C. The shape of the endothermic melting peak of the IND-2 compound is similar to the IND-1 peak and also indicates the high purity of the obtained derivative (Figure 9d, Figure 10d, Figure 11d and Figure 12d, green curves; Table 6) [21,22]. A clear exothermic peak was recorded on the c-DTA curve of the IND-2 derivative with a maximum of 266.3 °C. This peak is related to the thermal decomposition of the compound.

**Figure 13:**
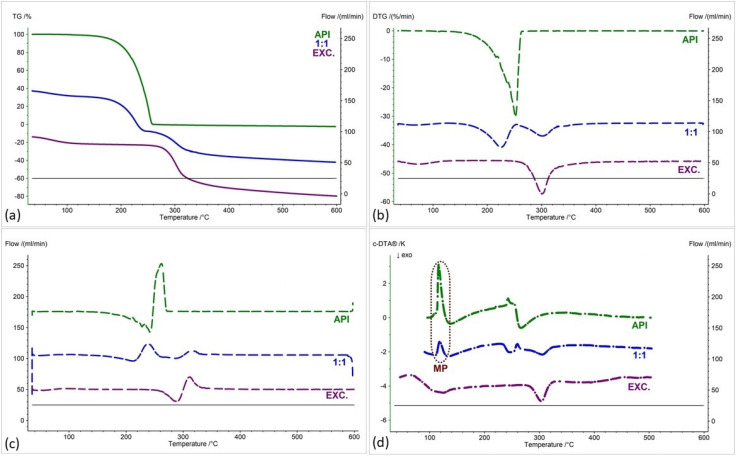
(**a**) TG, (**b**) DTG, (**c**) D2TG and (**d**) c-DTA curves of IND-2 (API), chitosan (EXC.) and binary mixture of API and excipient in a weight ratio of 1:1. MP—melting point.

**Figure 14:**
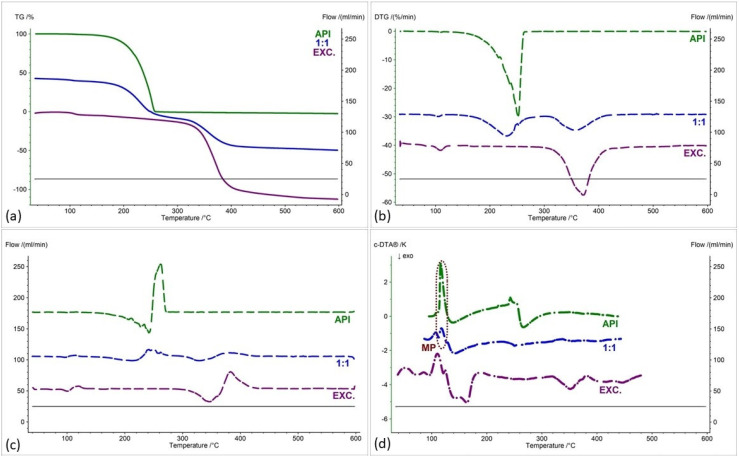
(**a**) TG, (**b**) DTG, (**c**) D2TG and (**d**) c-DTA curves of IND-2 (API), magnesium stearate (EXC.) and binary mixture of API and excipient in a weight ratio of 1:1. MP—melting point.

In the case of the IND-2 mixture with the tested excipients, no significant shift in the onset of the decomposition temperature and no significant change in the course of the thermogravimetric curve were observed compared to pure IND-2 (Figure 9, Figure 10, Figure 11, Figure 12, Figure 13 and Figure 14, Table 4). Among the tested mixtures in a weight ratio of 1:1, the mix of IND-2 with starch showed the most significant reduction in temperature at the beginning of decomposition (by 26.6 °C) compared to pure IND-2 (Figure 12, Table 4). Therefore, for the mixture of IND-2 and starch from corn, additional mixtures were prepared in weight ratios of 2:1 and 1:2. The shape of the recorded thermogravimetric curves of IND-2 mixtures with starch in various weight ratios showed that as the amount of excipient in the mixture increases, a separate peak originating from starch appears (Figure 12, Table 4 and Table 5). The appearance of the peak is not accompanied by a shift of the peak originating from the active substance. This proves the compatibility of the two substances [18,19,28,36,37,38,39]. Moreover, the analysis of c-DTA curves showed no change or shift in the melting temperature recorded for the mixture compared to pure IND-2 (Figure 12d, Table 6). This phenomenon additionally confirms the compatibility of the tested mixtures regardless of the weight ratio used to prepare them.

**Figure 15:**
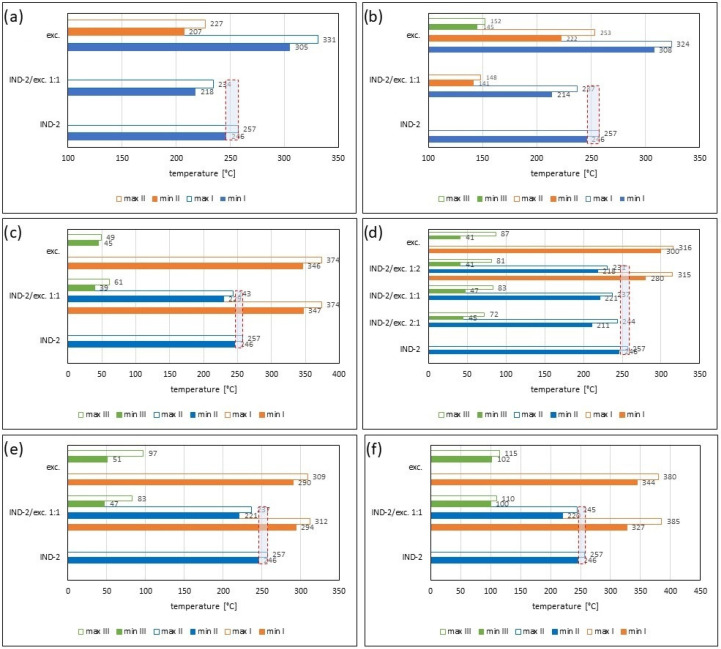
Temperature ranges recorded on D2TG curves for IND-2 (API): (**a**) glucose anhydrous, (**b**) lactose monohydrate, (**c**) microcrystalline cellulose, (**d**) starch from corn, (**e**) chitosan and (**f**) magnesium stearate; binary mixtures of API and excipients in weight ratios of (**a**–**f**) 1:1 and (**d**) 2:1 and 1:2. **Min**—initial temperature peak recorded on the D2TG curve; **Max**—final temperature peak recorded on the DTG curve; **I**, **II**, **III**—subsequent stages of decomposition. The blue boxes with red frames show the temperature ranges of the decomposition stage recorded for IND-2 and its location in the decomposition steps of the IND-2–excipient mixtures.

The recorded thermogravimetric curves for the mixtures of IND-2 with the tested excipients show the course of pure IND-2 (Figure 9a–c and Figure 10a–c) or contain separated thermal events originating from IND-2 and the excipients (Figure 11a–c, Figure 12a–c, Figure 13a–c, Figure 14a–c and Figure 15). The recorded c-DTA curves also confirm the compatibility of these mixtures (Figure 9d, Figure 10d, Figure 11d, Figure 12d, Figure 13d and Figure 14d, Table 6). As in the case of IND-1, we did not observe any shift in the melting point of the IND-2 used in the prepared mixtures. 

It is worth emphasizing that the IND-1 and IND-2 studies analyzed compatibility with chitosan, which has not been used as an excipient in tablets containing ACEIs so far (Table S1 in the Supplementary Materials). Chitosan is a valued excipient due to its biocompatibility properties and the increasing solubility of poorly water-soluble APIs, including drugs, used to treat cardiovascular diseases [40,41,42]. Sip et al. indicated the significant potential of using chitosan as an excipient in modern drug delivery systems containing carvediol [40]. Moreover, a review of the published literature showed that chitosan has low oral toxicity [42,43].

### 2.3. Colorimetric Analysis

In the work, colorimetric analysis in the CIE L*a*b* space system was used. Moreover, using the recorded values of L*, a* and b*, the ΔE parameter and browning index (BI) were determined (Table 7). The ΔE parameter determines the numerical color change between the two compared samples and takes higher values when the color difference is more visible [44,45]. 

The color of the samples of pure IND-1 and IND-2 substances and their mixtures with the tested excipients was assessed. In order to assess possible changes in color that could indicate a lack of compatibility, the tested samples were stored for 14 days under room and stress conditions. 

The tests showed that the stress conditions caused a noticeable color change in the IND-1 mixtures with glucose anhydrous, lactose monohydrate, microcrystalline cellulose, chitosan and magnesium stearate. In turn, in the cases of mixtures of IND-2 with lactose monohydrate, microcrystalline cellulose, starch from corn and chitosan, a color change was observed. Therefore, in terms of color change, the most appropriate potential excipients in formulations containing IND-1 are starch, and, in the case of IND-2, they are glucose anhydrous and magnesium stearate. No color change was observed for these mixtures even after storage under stress conditions. However, it should be remembered that colorimetric tests are of a screening nature because the storage conditions used are very drastic (temp. = 60 °C, RH = 75%), and the color change does not occur under room conditions (temp. = 25 °C, RH = 45%). 

Lactose and glucose have a reactive carbonyl group that may be responsible for the chemical incompatibility and instability of lactose, glucose and a mixture of APIs with these sugars. This may be due to reactions characteristic of the carbonyl group. An example is the Maillard reaction.

In the case of mixtures showing the possibility of a Maillard reaction, the browning index was additionally calculated. As demonstrated by numerous scientific publications, Maillard reactions are accompanied by color reactions [18,46,47,48,49]. These changes are related to the yellowing or browning of a sample [18,46,47,48,49,50]. It was also shown that colorimetric analysis can be a very valuable method for the identification and kinetics of the Maillard reaction [47,48,50]. The browning index was calculated for IND-1 mixtures with glucose and lactose stored for 14 days in room and stress conditions. In both cases, storage in stress conditions caused an increase in the BI value. In the case of the IND-1 mixture with glucose, the change in BI was from 76.54 to 79.25. In the case of the mixture with lactose, this change was from 76.58 to 77.68. The obtained values indicate the browning of the samples and may confirm the occurrence of the Maillard reaction.

### 2.4. UV–Vis Spectrophotometry

In this work, UV absorbance spectra were determined for the tested derivatives of IND-1 and IND-2 and for mixtures of the tested derivatives with the tested excipients. UV spectra recorded for the mixtures stored under stress conditions (60 °C/75%RH/14 days) were evaluated. 

**Figure 16:**
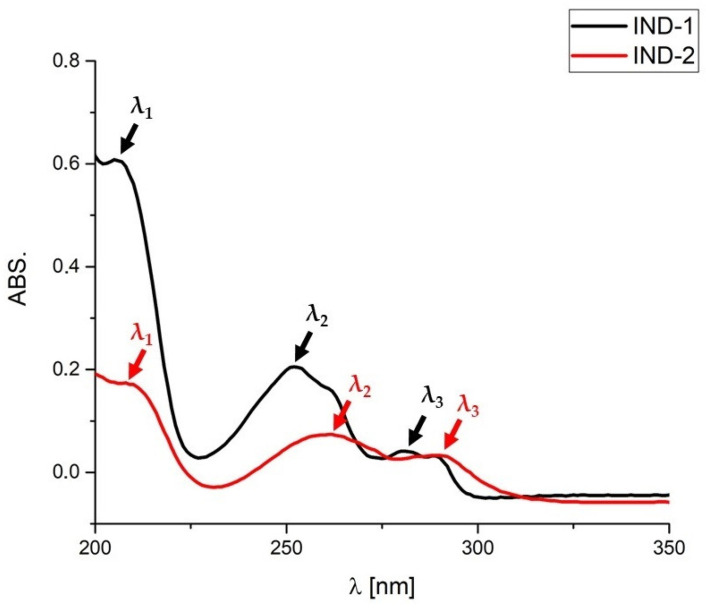
UV spectra of IND-1 and IND-2. λ_1_, λ_2,_ λ_3_—wavelengths at which absorption maxima occurred.

For pure derivatives of IND-1 and IND-2, three absorbance maxima were recorded, which are shown in Figure 16. All tested mixtures showed absorbance maxima that were the same as or very close to those of pure IND-1 and IND-2 (Table 8). 

In the case of the mixture of IND-1 with starch from corn and magnesium stearate, a slight bathochromic shift was observed for λ_2_ and a hypsochromic shift for λ_3_. 

In turn, for the IND-2 mixtures, slight shifts were observed for the mixtures with starch from corn, chitosan and magnesium stearate. In the case of λ_1_, a hypsochromic shift was recorded for starch from corn and chitosan and a bathochromic shift for magnesium stearate. In the case of λ_2_, a hypsochromic shift was also recorded for starch and chitosan. In the case of λ_3_, a hypsochromic shift was recorded only for the mixture with chitosan. 

As reported by the studies, the UV–Vis method was previously successfully used to assess the effect of UV radiation on the degradation of quercetin [51] and to assess the concentration of glibenclamide in commercial drugs and its in vitro interactions with other groups of drugs, such as NSAIDs and fluoroquinolone antibiotics [52]. However, the studies conducted in our work did not show significant changes in the UV spectra that could clearly determine the interaction between IND-1 and IND-2 and the tested excipients.

### 2.5. FTIR Measurements

**Figure 17:**
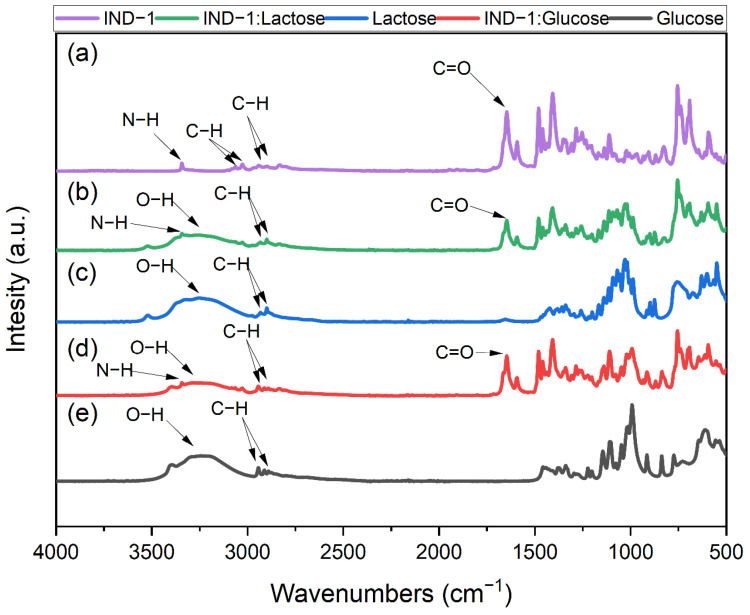
FTIR analysis of (**a**) pure IND-1, (**b**) IND-1–lactose monohydrate, (**c**) lactose monohydrate, (**d**) IND-1–glucose anhydrous and (**e**) glucose anhydrous.

The FTIR spectrum of pure IND-1 (Figure 17a) displays a band at ~3340 cm⁻¹, attributed to the N-H stretch of the secondary amine [53]. Additionally, two peaks at ~3066 and ~3027 cm⁻¹ [54] are observed (assigned to aromatic C-H stretching). The peaks at 2937 and 2900 cm⁻¹ correspond to aliphatic C-H stretching vibrations [55], while amide carbonyl (C=O) stretching vibrations are indicated by a peak at ~1647 cm⁻¹ [55].

Figure 17b presents the spectrum of IND-1–lactose monohydrate. The N-H bond appears at ~3340 cm⁻¹ [53], followed by a broad band representing O-H stretching vibrations [56]. The two peaks at ~2932 and ~2896 cm⁻¹ correspond to C-H stretching vibrations [55], with amide C=O stretching vibrations also observed at ~1647 cm⁻¹ [55].

Figure 17c shows the FTIR spectrum of lactose monohydrate, highlighting major peaks associated with O-H (a broad band at ~3400–~3000 cm⁻¹) [54] and C-H stretching vibrations at ~2930 and ~2897 cm⁻¹ [55].

Figure 17d shows the spectrum of IND-1–glucose anhydrous; the first peak at ~3340 cm⁻¹ represents N-H stretch [53]. Next to it, a broad peak appears, representing O-H [56]. Bands identified as C-H are observed at ~2940 and ~2890 cm⁻¹ [55], while the peak at ~1647 cm⁻¹ as described earlier represents C=O [55].

The last spectrum in Figure 17e shows the FTIR analysis of glucose anhydrous, featuring characteristic O-H peaks (a broad band at 3400–3000 cm⁻¹) [56] and two peaks at 2940 and 2890 cm⁻¹ corresponding to C-H stretching vibrations [55].

**Figure 18:**
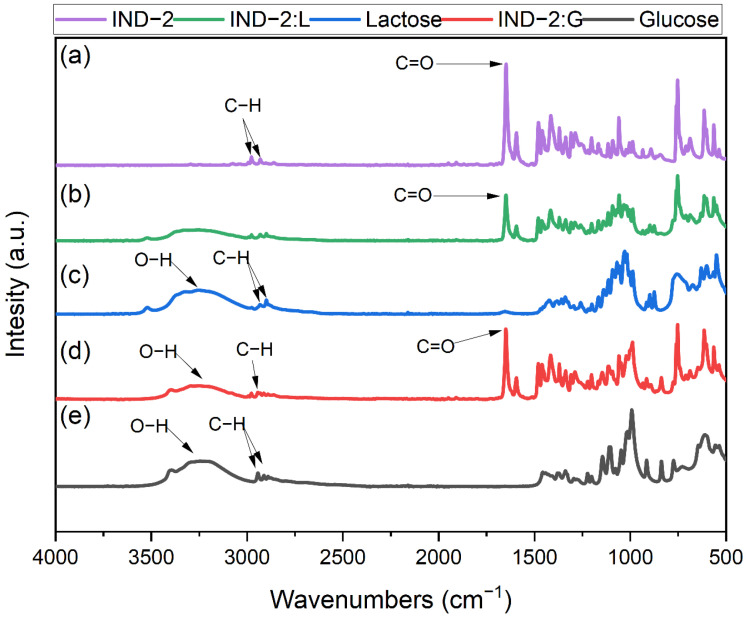
FTIR analysis of (**a**) pure IND-2, (**b**) IND-2–lactose monohydrate, (**c**) lactose monohydrate, (**d**) IND-2–glucose anhydrous and (**e**) glucose anhydrous.

Figure 18a illustrates the FTIR analysis of the pure IND-2. In the spectrum, the following characteristic peaks can be observed: at ~2979 and ~2932 cm^−1^, the C-H bond bands are identified [55]. In addition, at ~1647 cm^−1^ [55], the vibration associated with the C=O group is visible. Figure 18b shows the spectrum of IND-2–lactose monohydrate; the spectrum shows a broad band representative of O-H followed by two bands at ~2979 and ~2932 cm^−1^ that identify C-H stretching, then at ~1647 cm^−1^, C=O groups are identified. For the analysis of lactose monohydrate and glucose anhydrous (Figure 18c,e), refer to the above text. Figure 18d refers to IND-2–glucose anhydrous, a broad band that characterizes the O-H groups, followed by C-H (~2979 and ~2932 cm^−1^), while at ~1647 cm^−1^, the C=O vibration is visible.

The FTIR analysis indicates that excipients such as lactose monohydrate and glucose anhydrous did not alter the FTIR spectra of IND-1 and IND-2. The observed differences from pristine materials likely resulted from a mixture of IND-1 or IND-2 with the excipients, rather than an interaction between them. These results are consistent with previous analyses, such as colorimetric and UV–Vis analyses, which showed no significant interactions except under extreme storage conditions. Therefore, FTIR analysis proves to be a reliable method for evaluating these types of interactions.

For better readability, Table 9 summarizes the compatibility results obtained with the analytical techniques used in the work.

Our research focused mainly on the use of thermal analyses (TGA and c-DTA) to assess the compatibility of the tested compounds with excipients. Thermal methods were selected due to their advantages such as speed and their requiring only small sample volumes for measurement, as well as the low costs of performing the tests. Moreover, as reported by numerous scientific studies, thermal analyses, including TGA and c-DTA, are successfully used to assess the compatibility of APIs with excipients [18,19,28,36,37,38,39]. However, these methods also have limitations. They are particularly evident in the case of substances that, under the influence of high temperature, create incompatibilities that do not occur at room temperature. In such cases, the use of non-thermal methods is necessary. In our work, among the non-thermal technics, we used the FTIR, UV–Vis and colorimetric methods. The use of non-thermal methods and the mutual complementation of the applied thermal methods (TGA and c-DTA) allowed us to resolve doubts regarding the compatibility of the IND-1 compound with glucose anhydrous and lactose monohydrate.

Based on the conducted studies, it can be concluded that the incompatibility of IND-1 with glucose anhydrous and lactose monohydrate occurs only when the mixture is stored at higher temperatures. This is particularly visible in the case of colorimetric studies. For samples stored for 14 days in room conditions, no color change was noted for any CIE L*a*b* parameter. However, in the case of samples stored for 14 days in stress conditions (temp. = 60 °C, RH = 75%), color changes were visible. Additionally, FTIR spectra of the IND-1 starting mixture with glucose anhydrous and lactose monohydrate did not show any changes.

## 3. Materials and Methods

### 3.1. ACEI Indole Derivatives Used in the Research

**2-chloro-1-(indolin-1-yl)propoan-1-one (1, IND-2)**

In a round-bottom flask, indoline (10.12 mL, 50 mmol) was dissolved in THF (50 mL), then 2-chloropropionyl chloride (4.38 mL, 55 mmol) was added dropwise to the solution for 30 min, after which the reaction was mixed at room temperature for 30 min. After that time, the reaction mixture was poured out on water–ice (1:1 200 mL). The white precipitate was treated and purified via column chromatography, using dichlorometane–metalone (50:1) as eluent, to give **1**, IND-2 (9.43 g, yield: 90%).

**2-chloro-1-(indolin-1-yl)ethan-1-one (2)**

In a round-bottom flask, indoline (10.12 mL, 50 mmol) was dissolved in THF (50 mL), then 2-chloropropionyl chloride (5.38 mL, 55 mmol) was added dropwise to the solution for 30 min, after which the reaction was mixed at room temperature for 30 min. After that time, the reaction mixture was poured out on water–ice (1:1 200 mL). The white precipitate was treated and purified via column chromatography, using dichlorometane–methanol (50:1) as eluent, to give **2** (8.51 g, yield: 87%).

**2-(benzylamino)-1-(2,3-dihydroindol-1-yl)ethanone (3, IND-1)**

Compound 2 (2.9 g 15 mmol) was dissolved in THF, then K_2_CO_3_ (3.1 g, 22.5 mmol), KI (3.0 g, 18 mmol) and benzylamine (1.65 mL, 15 mmol) were added. The reaction mixture was stirred at reflux for 24 h and then poured out on an ice–water mixture (1:1, 200 mL). The yellow precipitate was filtrated. Column flash chromatography in dichlorometane–methanol (50:1) led to a yellow solid, **3** (3.3 g, yield: 83%). Mp 92–94 °C. R_f_ 0.24 (CH_2_Cl_2_:CH_3_OH 50:1). UV–Vis (CH_2_Cl_2_): λ_max_, nm (logε) 310 (4.2), ^1^H NMR (500 MHz; DMSO- d_6_): δH, ppm 3.06–3.14 (m, 2H), 3.46 (s, 1H), 3.60–3.67 (m, 1H) 3.77 (s, 1H), 4.02 (m, 3H), 6.97–7.00 (m, 1H), 7.12–7.17 (m, 1H), 7.21–7.27 (m, 3H), 7.29–7.39 (m, 4H), 8.09 (d, 1H). 13C NMR (125 MHz; DMSO- d_6_): δC, ppm 170.1; 169.2; 143.4; 141.0; 139.3; 132.0; 129.3; 128.7; 128.4; 127.5; 127.4; 127.1; 125.3; 116.5, 116.3, 58.1; 56.5; 53.1; 51.6; 47.1; 46.7; 28.0. MS ES m/z 267 [M + H^+^]. HRMS m/z found: 267.1489 [M + H]^+^ C_17_H_19_N_2_O requires 267.14973 [M + H]^+^.

### 3.2. Excipients Used in the Research

All excipients used in the experiment were purchased commercially from Sigma-Aldrich (Merck, Rahway, NJ, USA). Glucose monohydrate (catalogue number G8270), formula: C_6_H_12_O_6_, purity of 99.5%, mass: 180.16 g/mol; lactose monohydrate (catalogue number 107656), formula: C_12_H_22_O_11_ · H_2_O, purity of 99.5%, mass: 360.32 g/mol; microcrystalline cellulose (catalogue number Y0002021), formula: (C_6_H_10_O_5_)_n_, purity of 99.5%; starch from corn (catalogue number S4180), formula: (C_6_H_10_O_5_)_n_, purity of 99.5%,; chitosan (catalogue number Y0000104), formula: C_12_H_24_N_2_O_9_, purity of 99.5%,; magnesium stearate (catalogue number PHR1363), formula C_36_H_70_MgO_4_, purity of 99.5%, molecular weight: 591.27 g/mol.

### 3.3. Performing Thermogravimetric Analyses of TG, DTG and c-DTA

Thermogravimetric analyses were performed using a thermogravimeter, the TG 209 F3 Tarsus, produced by Netzsch (Selb, Germany). For the tested samples, thermogravimetric dynamic measurements were performed. Dynamic TG, DTG, D2TG and c-DTA analyses were performed in an atmosphere of inert gas (nitrogen) in the temperature range of 35–600 °C, with a heating rate of 10 k/min. The mass of each sample for determining thermogravimetric curves was 10 mg.

### 3.4. Analysis of Mixtures under Room and Stress Conditions

IND-1 and IND-2 mixtures with the tested excipients were subjected to stress conditions by storing them in an incubator with controlled temperature and relative humidity. The mixtures were placed on Petri dishes and stored at a temperature of 60 °C (±0.5 °C) and a relative humidity (RH) of 75% (±5%) for a duration of 14 days [1,57]. For comparison purposes, IND-1 and IND-2 mixtures with the tested excipients were simultaneously stored at room conditions. The mixtures were placed on Petri dishes and stored at a temperature of 25 °C (±0.5 °C) and a relative humidity (RH) of 45% (±5%) for a duration of 14 days.

In both cases, the samples were stored in a professional hot-air oven produced by the Memmert company (Schwabach, Germany) with air circulation, which maintained constant parameters.

### 3.5. Performing Colorimetric Analysis

Colorimetric analysis in the CIE L*a*b* color system was performed. Analyses of color parameters (L*, a*, b*) and total color difference parameters (ΔE) were performed for pure IND-1, the IND-2 compound and the binary mixture of IND-1 and IND-2 with tested excipients in a ratio of 1:1. The mixtures were tested after 14 days of storage under room conditions. For this purpose, the NH 310 colorimeter produced by the 3 nh Company (China) was used.

Parameter L* refers to the lightness of tested samples. Parameter a* refers to the redness of samples. Parameter b* refers to the yellowness of samples [44,45]. The interpretation of the color change using the ΔE parameter is shown in Table 10.

The browning index (BI) was calculated for the IND-1 mixtures with glucose anhydrous and lactose monohydrate according to Formula (1), using the parameters obtained for the CIE L*a*b* measurements [58]:

BI = 100[(x − 0.31)]/0.172,(1)

where parameter x was calculated according to Formula (2):

x = (a* + 1.75 L*)/(5.645 L* + a* − 3.012 b*),(2)

All measurements were performed six times for each sample. The received values were averaged (±SDs). The one-way ANOVA test was used to assess statistical significance. Statistical significance was assumed with *p* < 0.05. Statistical analysis was performed using the statistical software produced by TIBCO Software Inc. (Palo Alto, CA, USA).

### 3.6. UV–Vis Analysis

UV absorbance spectra of the IND-1 and IND-2 derivatives and the binary mixtures of IND-1 and IND-2 with the tested excipients in a ratio of 1:1 were recorded. The mixtures were tested after 14 days of storage under stress conditions (temperature = 60 °C, relative humidity = 75%). For this purpose, 10 mg of each of the samples was dissolved in 100 mL of purified water. The samples were then mixed and poured into a quartz cuvette, which was placed in a UV–Vis spectrophotometer. UV absorbance spectra were recorded in the wavelength range from 200 nm to 350 nm. The Thermo Genesys 10S UV–Vis spectrophotometer produced by Thermo Scientific (Waltham, MA, USA) was used. To obtain and analyze the UV spectra, VisionLite Software (Thermo Scientific Company, Waltham, MA, USA) and Origin 2016 (OriginLab Company, Northampton, MA, USA) were used.

### 3.7. FTIR Meansuremenets

Fourier-transform infrared spectroscopy spectra (4 cm^−1^ resolution at 4000–500 cm^−1^) were obtained with a germanium ATR crystal (NICOLET IZ10; Thermo Scientific, Waltham, MA, USA) with a horizontal ATR attachment with a single reflection angle of 45°.

## 4. Conclusions

The studies showed the usefulness of the thermal methods used (a combination of TGA and c-DTA) for assessing the compatibility of newly synthesized ACEI derivatives with selected excipients because both methods complement each other very well.

In the case of the presence of an amino group in the API and the use of reducing sugars as excipients, we can expect the Maillard reaction to occur in the case of higher temperatures. Therefore, thermal analysis can give a positive result of the interaction, which occurs only when the drug is exposed to higher temperatures. In such cases, the use of FTIR analysis allows for making a final decision on the type of interaction. Moreover, in this case, the studies also showed the usefulness of colorimetric analysis in the CIE L*a*b* system and the ΔE and BI parameters obtained from the L* a* and b* data for identifying interactions occurring at elevated temperatures. The results obtained for the IND-1 mixtures with lactose monohydrate and glucose anhydrous indicate a visible change in the color of the samples stored only at elevated temperatures, which confirms the possibility of the Maillard reaction.

However, when selecting excipients when designing a drug form, it should be taken into account that unfavorable thermal conditions can affect the drug during its improper storage, during transport or at the patient’s home. Therefore, lactose and glucose should be cautiously used as excipients in drugs containing amine groups.

## Figures and Tables

**Table 1:** Parameters of recorded TG curves for IND-1, pure excipients and mixtures of IND-1 with individual excipients.

Samples	Ratio (W:W)	Onset [°C]	Mid [°C]	Inflection [°C]	End [°C]	Weight Loss [%]
IND-1		275.5	306.0	304.9	336.4	−76.68
Glucose anhydrous		279.3	311.0	318.7	343.4	−70.65
IND-1:EXC.	2:1	247.0	288.8	315.5	340.4	−58.14
	1:1	180.2	268.7	197.4	268.8	−60.93
	1:2	174.3	213.2	186.2	229.3	−54.49
Lactose monohydrate		259.2	299.9	310.0	338.2	−48.63
IND-1:EXC.	2:1	270.3	310.6	321.6	350.0	−57.77
	1:1	187.7	296.4	201.9	278.6	−61.03
	1:2	192.4	252.1	206.4	250.2	−45.99
Microcrystalline cellulose		339.7	356.6	357.8	373.3	−81.0
IND-1:EXC.	1:1	266.2	309.8	320.1	352.8	−69.26
Starch from corn		297.8	309.6	309.2	321.4	−61.45
IND-1:EXC.	1:1	260.0	296.0	290.9	333.2	−69.60
Chitosan		283.0	302.4	300.4	322.3	−42.60
IND-1:EXC.	1:1	269.2	299.2	301.6	329.6	−52.16
Magnesium stearate		339.8	365.5	372.0	391.1	−84.73
IND-1:EXC.	1:1	257.8	295.7	288.8	342.3	−55.0

**Table 2:** Parameters of recorded DTG curves for IND-1, pure excipients and mixtures of IND-1 with individual excipients. **Bold**—decomposition step related to IND-1.

Samples	Ratio (W:W)	Stage [First, Second, Third, Fourth] Peak [°C] Mass Change [%/min.]					
IND-1		-	-	-	**First** **305.4** **13.61**	-	-
IND-1:EXC.	2:1	-	First 122.8 1.30	Second 184.3 4.47	**Third** **313.6** **6.65**	-	-
	1:1	-	First 118.0 0.60	Second 197.7 7.68	**Third** **314.9** **5.68**	-	-
	1:2	-	First 126.1 0.67	Second 187.1 10.79	**Third** **308.7** **3.84**	-	-
Glucose anhydrous		-	-	First 219.9 3.74	Second 319.2 11.61	-	-
IND-1:EXC.	2:1	First 134.1 1.37	Second 145.1 1.37	Third 194.6 5.10	**Fourth** **322.2** **8.52**	-	-
	1:1	First 137.5 1.54	Second 151.8 2.01	Third 202.6 8.60	**Fourth** **318.2** **7.20**	-	-
	1:2	First 139.9 1.68	Second 149.9 1.97	Third 205.8 9.95	**Fourth** **309.3** **4.23**	-	-
Lactose monohydrate		-	First 148.6 3.24	Second 236.1 5.84	Third 308.2 7.49	-	-
IND-1:EXC.	1:1	-	-	-	**First** **320.5** **9.43**	-	-
Microcrystalline cellulose		-	-	First 47.7 0.78	Second 359.8 25.68	-	-
IND-1:EXC.	1:1	-	-	First 68.3 0.64	**Second** **289.3** **10.05**	-	-
Starch from corn		-	-	First 71.7 2.05	Second 309.0 26.14	-	-
IND-1:EXC.	1:1	-	-	First 65.5 1.24	**Second** **302.1** **9.33**	-	-
Chitosan		-	-	First 72.6 1.39	Second 301.1 11.62	-	-
IND-1:EXC.	1:1	First 107.0 0.79	-	-	**Second** **287.0** **7.66**	Third 374.2 6.63	Fourth 456.1 3.49
Magnesium stearate		First 109.3 1.89	-	-	-	Second 372.0 17.59	-

**Table 3:** Parameters of recorded c-DTA curves for IND-1, pure excipients and mixtures of IND-1 with individual excipients. **En.**—endothermic reaction, **Ex.**—exothermic reaction, **melting point**—bolded.

Tested Samples	Parameter	Peak			
		I	II	III	IV
IND-1	Onset [°C]	**76.9**	162.4	-	-
	Peak [°C]	**81.5**	172.2	-	-
	Type of reaction	**En.**	Ex.	-	-
	Area [K*s]	**53.9**	33.5	-	-
Glucose anhydrous	Onset [°C]	154.2	226.4	324.2	-
	Peak [°C]	157.5	257.8	337.7	-
	Type of reaction	En.	Ex.	Ex.	-
	Area [K*s]	209.4	232.5	38.4	-
IND-1:EXC. 2:1	Onset [°C]	**73.8**	127.3	-	-
	Peak [°C]	**78.6**	177.0	-	-
	Type of reaction	**En.**	Ex.	-	-
	Area [K*s]	**21.6**	79.8	-	-
IND-1:EXC. 1:1	Onset [°C]	**69.2**	138.5	193.8	-
	Peak [°C]	**80.1**	169.8	207.3	-
	Type of reaction	**En.**	Ex.	Ex.	-
	Area [K*s]	**19.3**	123.2	52.1	-
IND-1:EXC. 1:2	Onset [°C]	**70.0**	144.6	187.7	-
	Peak [°C]	**80.1**	167.3	197.6	-
	Type of reaction	**En.**	Ex.	Ex.	-
	Area [K*s]	**8.4**	75.4	25.3	-
Lactose monohydrate	Onset [°C]	145.8	208.8	233.6	-
	Peak [°C]	150.2	214.8	261.9	-
	Type of reaction	En.	En.	Ex.	-
	Area [K*s]	59.2	122.2	161.9	-
IND-1:EXC. 2:1	Onset [°C]	**73.5**	125.5	149.6	-
	Peak [°C]	**79.1**	133.7	187.2	-
	Type of reaction	**En.**	En.	Ex.	-
	Area [K*s]	**33.9**	72.3	52.4	-
IND-1:EXC. 1:1	Onset [°C]	**73.3**	127.0	192.8	-
	Peak [°C]	**79.2**	142.2	207.5	-
	Type of reaction	**En.**	En.	Ex.	-
	Area [K*s]	**30.9**	98.9	84.3	-
IND-1:EXC. 1:2	Onset [°C]	**74.1**	129.5	198.6	-
	Peak [°C]	**78.6**	149.9	214.7	-
	Type of reaction	**En.**	En.	Ex.	-
	Area [K*s]	**15.5**	122.9	93.1	-
Microcrystalline cellulose	Onset [°C]	41.1	358.0	-	-
	Peak [°C]	50.1	382.5	-	-
	Type of reaction	En.	Ex.	-	-
	Area [K*s]	46.5	198.7	-	-
IND-1:EXC. 1:1	Onset [°C]	-	**74.3**	162.3	285.6
	Peak [°C]	46.3	**79.3**	178.8	331.0
	Type of reaction	En.	**En.**	Ex.	Ex.
	Area [K*s]	7.1	**23.2**	10.7	48.7
Starch from corn	Onset [°C]	42.0	283.0	-	-
	Peak [°C]	57.6	308.5	-	-
	Type of reaction	En.	Ex.	-	-
	Area [K*s]	157.2	512.7	-	-
IND-1:EXC. 1:1	Onset [°C]	-	**73.7**	162.5	277.5
	Peak [°C]	48.1	**78.1**	174.0	294.9
	Type of reaction	En.	**En.**	Ex.	Ex.
	Area [K*s]	12.9	**11.1**	32.3	23.4
Chitosan	Onset [°C]	45.1	285.0	-	-
	Peak [°C]	60.1	303.5	-	-
	Type of reaction	En.	Ex.	-	-
	Area [K*s]	65.7	141.3	-	-
IND-1:EXC. 1:1	Onset [°C]	-	**76.1**	171.4	279.6
	Peak [°C]	44.4	**79.7**	185.7	302.7
	Type of reaction	En.	**En.**	Ex.	Ex.
	Area [K*s]	91.2	**7.1**	4.1	53.2
Magnesium stearate	Onset [°C]	73.3	93.2	321.7	-
	Peak [°C]	85.0	110.5	352.0	-
	Type of reaction	En.	En.	Ex.	-
	Area [K*s]	6.9	84.8	134.6	-
IND-1:EXC. 1:1	Onset [°C]	**76.9**	99.8	162.2	361.9
	Peak [°C]	**81.0**	108.9	177.4	386.7
	Type of reaction	**En.**	En.	Ex.	Ex.
	Area [K*s]	**16.3**	30.5	21.5	126.4

**Table 4:** Parameters of recorded TG curves for IND-2, pure excipients and mixtures of IND-2 with individual excipients.

Samples	Ratio (W:W)	Onset [°C]	Mid [°C]	Inflection [°C]	End [°C]	Weight Loss [%]
IND-2		224.7	238.9	252.1	257.3	−92.08
Glucose anhydrous		279.3	311.0	318.7	343.4	−70.65
IND-2:EXC.	1:1	199.2	220.4	227.7	243.3	−61.59
Lactose monohydrate		259.2	299.9	310.0	338.2	−48.63
IND-2:EXC.	1:1	204.5	226.2	227.1	246.6	−57.50
Microcrystalline cellulose		339.7	356.6	357.8	373.3	−81.0
IND-2:EXC.	1:1	210.0	200.7	237.7	191.4	−46.2
Starch from corn		297.8	309.6	309.2	321.4	−61.45
IND-2:EXC.	2:1	202.7	226.4	235.1	249.7	−75.81
	1:1	198.1	223.7	226.5	248.9	−57.41
	1:2	261.3	287.0	304.5	323.0	−65.19
Chitosan		283.0	302.4	300.4	322.3	−42.60
IND-2:EXC.	1:1	199.6	204.6	227.6	209.7	−43.23
Magnesium stearate		339.8	365.5	372.0	391.1	−84.73
IND-2:EXC.	1:1	198.8	251.6	230.7	295.9	−67.96

**Table 5:** Parameters of recorded DTG curves for IND-2, pure excipients and mixtures of IND-2 with individual excipients. **Bold**—decomposition step related to IND-2.

Samples	Ratio (W:W)	Stage [First, Second, Third, Fourth] Mass Change [%/min.] Peak [°C]		
IND-2		-	**First** **252.1** **29.72**	-
IND-2:EXC.	1:1	-	**Third** **227.2** **15.28**	-
Glucose anhydrous		First 219.9 3.74	-	Second 319.2 11.61
IND-2:EXC.	1:1	First 144.2 1.92	**Second** **228.7** **15.23**	
Lactose monohydrate		First 148.6 3.24	Second 236.1 5.84	Third 308.2 7.49
IND-2:EXC.	1:1	First 46.4 0.36	**Second** **236.9** **14.60**	Third 359.9 9.72
Microcrystalline cellulose		First 47.7 0.78	-	Second 359.8 25.68
IND-2:EXC.	2:1	First	**Second**	-
		65.5	**232.9**	
		0.62	**17.64**	-
	1:1	First 65.8 1.32	**Second** **226.1** **13.76**	-
	1:2	First 57.4 1.69	**Second** **225.5** **9.47**	Third 305.2 10.14
Starch from corn		First 71.7 2.05	-	Second 309.0 26.14
IND-2:EXC.	1:1	First 67.9 1.01	**Second** **231.5** **10.74**	Third 302.8 5.49
Chitosan		First 72.6 1.39	-	Second 301.1 11.62
IND-2:EXC.	1:1	First 105.3 0.99	**Second** **231.4** **7.79**	Third 356.7 5.86
Magnesium stearate		First 109.3 1.89	-	Second 372.0 17.59

**Table 6:** Parameters of recorded c-DTA curves for IND-2, pure excipients and mixtures of IND-2 with individual excipients. **En.**—endothermic reaction, **Ex.**—exothermic reaction, **melting point**—bolded.

Tested Samples	Parameter	Peak			
		I	II	III	IV
IND-2	Onset [°C]	**114.4**	247.0	-	-
	Peak [°C]	**116.4**	266.3	-	-
	Type of reaction	**En.**	Ex.	-	-
	Area [K*s]	**121.5**	213.6	-	-
Glucose anhydrous	Onset [°C]	**154.2**	226.4	324.2	-
	Peak [°C]	**157.5**	257.8	337.7	-
	Type of reaction	**En.**	Ex.	Ex.	-
	Area [K*s]	**209.4**	232.5	38.4	-
IND-2:EXC. 1:1	Onset [°C]	**112.2**	149.0	230.2	-
	Peak [°C]	**117.0**	154.2	240.9	-
	Type of reaction	**En.**	En.	Ex.	-
	Area [K*s]	**57.4**	90.3	93.6	-
Lactose monohydrate	Onset [°C]	145.8	208.8	233.6	-
	Peak [°C]	150.2	214.8	261.9	-
	Type of reaction	En.	En.	Ex.	-
	Area [K*s]	59.2	122.2	161.9	-
IND-2:EXC. 1:1	Onset [°C]	**113.1**	140.1	196.8	227.6
	Peak [°C]	**118.0**	145.4	200.9	246.5
	Type of reaction	**En.**	En.	En.	Ex.
	Area [K*s]	**53.3**	27.1	95.0	116.2
Microcrystalline cellulose	Onset [°C]	41.1	358.0	-	-
	Peak [°C]	50.1	382.5	-	-
	Type of reaction	En.	Ex.	-	-
	Area [K*s]	46.5	198.7	-	-
IND-2:EXC. 1:1	Onset [°C]	-	**113.1**	241.7	361.4
	Peak [°C]	37.6	**118.3**	251.1	382.1
	Type of reaction	En.	**En.**	Ex.	Ex.
	Area [K*s]	28.8	**65.3**	82.3	59.9
Starch from corn	Onset [°C]	42.0	283.0	-	-
	Peak [°C]	57.6	308.5	-	-
	Type of reaction	En.	Ex.	-	-
	Area [K*s]	157.2	512.7	-	-
IND-2:EXC. 2:1	Onset [°C]	-	**112.5**	237.5	-
	Peak [°C]	53.4	**116.0**	253.3	-
	Type of reaction	En.	**En.**	Ex.	-
	Area [K*s]	72.4	**90.4**	90.1	-
IND-2:EXC. 1:1	Onset [°C]	-	**112.3**	233.2	-
	Peak [°C]	47.6	**116.8**	249.2	-
	Type of reaction	En.	**En.**	Ex.	-
	Area [K*s]	125.8	**51.9**	142.8	-
IND-2:EXC. 1:2	Onset [°C]	-	**113.4**	230.2	294.2
	Peak [°C]	38.5	**118.5**	238.6	318.8
	Type of reaction	En.	**En.**	Ex.	Ex.
	Area [K*s]	138.9	**29.3**	47.1	38.4
Chitosan	Onset [°C]	45.1	285.0	-	-
	Peak [°C]	60.1	303.5	-	-
	Type of reaction	En.	Ex.	-	-
	Area [K*s]	65.7	141.3	-	-
IND-2:EXC. 1:1	Onset [°C]	-	**111.0**	236.2	281.2
	Peak [°C]	39.7	**118.4**	244.0	304.8
	Type of reaction	En.	**En.**	Ex.	Ex.
	Area [K*s]	111.1	**30.9**	18.3	45.6
Magnesium stearate	Onset [°C]	73.3	93.2	321.7	-
	Peak [°C]	85.0	110.5	352.0	-
	Type of reaction	En.	En.	Ex.	-
	Area [K*s]	6.9	84.8	134.6	-
IND-2:EXC. 1:1	Onset [°C]	93.4	**111.6**	246.6	338.2
	Peak [°C]	107.2	**117.7**	250.2	360.1
	Type of reaction	En.	**En.**	Ex.	Ex.
	Area [K*s]	13.2	**18.6**	38.3	68.4

**Table 7:** Parameters of recorded colorimetry in the CIE L*a*b* system and the ΔE parameter. **GA**—glucose anhydrous, **LM**—lactose monohydrate, **MC**—microcrystalline cellulose, **SC**—starch from corn, **CH**—chitosan, **MS**—magnesium stearate. The results are presented as means ± SDs (n = 6). The results were considered statistically significant when *p* < 0.05.

Samples	Starting Sample			25 °C/45%RH/14 days				60 °C/75%RH/14 days			
	L* [±SD]	a* [±SD]	b* [±SD]	L* [±SD]	a* [±SD]	b* [±SD]	ΔE [±SD]	L* [±SD]	a* [±SD]	b* [±SD]	ΔE [±SD]
IND-1	63.38	10.81	30.58	62.78	9.20	29.04	**1.43**	62.94	10.20	30.57	**1.55**
	[±0.30]	[±0.08]	[±0.16]	[±0.20]	[±0.07]	[±0.09]	**[±0.30]**	[±0.21]	[±0.38]	[±0.25]	**[±0.38]**
IND-1/GA	62.20	10.54	30.01	61.88	9.32	29.02	**1.50**	59.73	10.30	29.64	**2.51**
	[±0.19]	[±0.17]	[±0.09]	[±0.20]	[±0.02]	[±0.15]	**[±0.20]**	[±0.35]	[±0.50]	[±0.40]	**[±0.50]**
IND-1/LM	59.70	9.92	28.90	60.02	8.82	28.18	**1.34**	61.35	10.35	30.01	**2.36**
	[±0.03]	[±0.03]	[±0.02]	[±0.32]	[±0.03]	[±0.02]	**[±0.32]**	[±0.32]	[±0.13]	[±0.17]	**[±0.32]**
IND-1/MC	59.46	9.83	28.73	60.78	9.69	28.91	**1.34**	63.31	10.49	30.54	**4.30**
	[±0.08]	[±0.01]	[±0.08]	[±0.94]	[±0.04]	[±0.03]	**[±0.94]**	[±0.07]	[±0.13]	[±0.04]	**[±0.13]**
IND-1/SC	61.34	10.35	29.38	61.06	9.52	29.70	**1.38**	62.55	10.19	29.82	**1.29**
	[±0.15]	[±0.02]	[±0.05]	[±0.02]	[±0.07]	[±0.11]	**[±0.15]**	[±0.40]	[±0.66]	[±0.44]	**[±0.66]**
IND-1/CH	62.07	10.50	29.80	61.09	10.34	29.61	**1.02**	59.33	9.41	28.28	**3.32**
	[±0.14]	[±0.19]	[±0.18]	[±0.14]	[±0.15]	[±0.22]	**[±0.22]**	[±0.41]	[±0.54]	[±0.44]	**[±0.54]**
IND-1/MS	60.98	10.21	28.89	61.64	10.09	28.77	**1.01**	63.64	8.99	28.29	**2.99**
	[±0.78]	[±0.30]	[±0.34]	[±0.35]	[±0.01]	[±0.25]	**[±0.78]**	[±0.67]	[±0.02]	[±0.32]	**[±0.78]**
IND-2	59.99	10.75	28.92	60.85	10.06	29.37	**1.19**	60.13	9.88	28.64	**0.93**
	[±0.17]	[±0.29]	[±0.21]	[±0.49]	[±0.07]	[±0.18]	**[±0.49]**	[±0.03]	[±0.04]	[±0.07]	**[±0.29]**
IND-2/GA	59.82	10.07	28.57	61.10	10.67	29.98 [±0.09]	**1.99**	60.60	9.48	28.56	**0.97**
	[±0.05]	[±0.18]	[±0.08]	[±0.11]	[±0.02]		**[±0.18]**	[±0.42]	[±0.14]	[±0.27]	**[±0.42]**
IND-2/LM	62.13	10.91	29.18	61.68	10.72	29.85	**0.83**	60.65	9.15	28.25	**2.49**
	[±0.86]	[±0.25]	[±0.20]	[±0.30]	[±0.18]	[±0.24]	**[±0.86]**	[±0.26]	[±0.14]	[±0.08]	**[±0.86]**
IND-2/MC	62.09	11.01	29.20	61.73	10.17	29.66	**1.55**	61.62	9.11	28.37	**2.13**
	[±0.04]	[±0.05]	[±0.07]	[±0.83]	[±0.33]	[±0.73]	**[±0.83]**	[±0.01]	[±0.02]	[±0.02]	**[±0.07]**
IND-2/SC	62.66	10.90	29.38	62.08	10.07	28.59	**1.26**	65.17	9.58	30.38	**3.00**
	[±0.04]	[±0.07]	[±0.18]	[±0.33]	[±0.09]	[±0.46]	**[±0.46]**	[±0.03]	[±0.03]	[±0.02]	**[±0.18]**
IND-2/CH	59.81	10.22	28.77	59.99	9.56	28.75	**1.27**	63.11	9.99	28.69	**3.30**
	[±0.23]	[±0.11]	[±0.13]	[±0.24]	[±0.03]	[±0.18]	**[±0.24]**	[±0.05]	[±0.03]	[±0.06]	**[±0.23]**
IND-2/MS	61.73	10.55	28.96	61.94	10.26	28.28	**1.48**	60.90	10.76	69.78	**0.92**
	[±0.24]	[±0.08]	[±0.13]	[±0.41]	[±0.32]	[±0.47]	**[±0.47]**	[±0.06]	[±0.03]	[±0.04]	**[±0.24]**

**Table 8:** Absorption maxima wavelengths for pure IND-1 and IND-2 compounds and their mixtures in a ratio of 1:1 with the tested excipients.

Samples	λ_1_ [nm]	λ_2_ [nm]	λ_3_ [nm]
IND-1	206	252	281
IND-1–glucose anhydrous	206	252	281
IND-1–lactose monohydrate	206	252	281
IND-1–microcrystalline cellulose	206	252	281
IND-1–starch from corn	206	257	279
IND-1–chitosan	206	252	281
IND-1–magnesium stearate	206	253	279
IND-2	208	260	290
IND-2–glucose anhydrous	208	260	290
IND-2–lactose monohydrate	208	260	290
IND-2–microcrystalline cellulose	208	260	290
IND-2–starch from corn	207	252	290
IND-2–chitosan	206	258	289
IND-2–magnesium stearate	209	260	290

**Table 9:** Compatibility of tested mixtures depending on the measurement method. **“+”**—compatible substances, **“-”**—no compatibility, **“0”**—no measurement, **GA**—glucose anhydrous, **LM**—lactose monohydrate, **MC**—microcrystalline cellulose, **SC**—starch from corn, **CH**—chitosan, **MS**—magnesium stearate.

Mixtures	TGA	c-DTA	FTIR	Colorimetry		UV–Vis
				Room Conditions	Stress Conditions	
IND-1/GA	-	+	+	+	-	+
IND-1/LM	-	+	+	+	-	+
IND-1/MC	+	+	0	+	-	+
IND-1/SC	+	+	0	+	+	+
IND-1/CH	+	+	0	+	-	+
IND-1/MS	+	+	0	+	-	+
IND-2/GA	+	+	+	+	+	+
IND-2/LM	+	+	+	+	-	+
IND-2/MC	+	+	0	+	-	+
IND-2/SC	+	+	0	+	-	+
IND-2/CH	+	+	0	+	-	+
IND-2/MS	+	+	0	+	+	+

**Table 10:** Interpretation of the color change using the ΔE parameter [45].

Value of Parameter ΔE	Color Change Visible to the Human Eye
<0.5	Not visible
From 0.5 to 1.5	Barely visible
From 1.5 to 3.0	Poorly visible
From 3.0 to 6.0	Visible
>6.0	Very visible

## Data Availability

The data that support the findings of this study are available from the corresponding author upon reasonable request.

## Supplementary Materials

The following supporting information can be downloaded at: https://www.mdpi.com/article/10.3390/ph17101323/s1. Figure S1: NMR data; Figure S2: HRMS spectra of IND-1 and IND-2; Figure S3: MS spectra of 2; Figure S4: UV-Vis spectra of IND-1 and IND-2; Table S1: The table presents commercial pharmaceutical preparations containing individual ACE inhibitors and the tested excipients.

## References

[1] Sznitowska M. (2017). Farmacja Stosowana.

[2] Kostowski W., Herman Z.S. (2016). Farmakologia.

[3] Tzourio C., Anderson C., Chapman N., Woodward M., Neal B., MacMahon S., Chalmers J. (2003). Effects of blood pressure lowering with perindopril and indapamide therapy on dementia and cognitive decline in patients with cerebrovascular disease. Arch. Intern. Med..

[4] Sink K.M., Leng X., Williamson J., Kritchevsky S.B., Yaffe K., Kuller L., Yasar S., Atkinson H., Robbins M., Psaty B. (2009). Angiotensin-converting enzyme inhibitors and cognitive decline in older adults with hypertension: Results from the Cardiovascular Health Study. Arch. Intern. Med..

[5] Juszczak A., Ramos P., Szczołko W., Pilawa B., Stanisz B. (2019). Can angiotensin-converting enzyme inhibitors interfere with the free radicals? Measurement of antioxidant capacity using DPPH radical reduction examined by UV-VIS method. Acta Pol. Pharm..

[6] Juszczak A., Ramos P., Szczołko W., Pilawa B., Stanisz B. (2020). Evaluation of antioxidant properties of angiotensin-converting enzyme inhibitors-interactions with free radicals model examined by EPR spectroscopy. Pharm. Pharmacol. Int. J..

[7] Janiec W. (2022). Farmakodynamika.

[8] Kollamaram G., Faucher A., Croker D.M., Walker G.M. (2018). Valvejet Technology for the Production of a Personalised Fixed Dose Combination of Ramipril and Glimepiride: An Investigative Study on the Stability of Ramipril. Pharm. Res..

[9] Paszun S., Stanisz B. (2013). Cilazapril decomposition kinetics and mechanism in the solid state versus stability of the other ester pro-drug angiotensin converting enzyme inhibitors. React. Kinet. Mech. Catal..

[10] Stanisz B. (2003). Evaluation of stability of enalapril maleate in solid phase. J. Pharm. Biomed. Anal..

[11] Stanisz B. (2004). The influence of relative humidity and temperature on stability of moexipril hydrochloride in solid phase. Acta Polon. Pharm..

[12] Polski A., Iwaniak K., Naleśniak M., Poleszak E. (2014). The excipients used in the non-coated tablests—A review. Med. Int. Revuo..

[13] Elder D.P., Kuentz M., Holm R. (2016). Pharmaceutical excipients—Quality, regulatory and biopharmaceutical considerations. Eur. J. Pharm. Scien..

[14] Bharate S.S., Bharate S.B., Bajaj A.N. (2010). Interactions and incompatibilities of pharmaceutical excipients with active pharmaceutical ingredients: A comprehensive review. J. Excip. Food Chem..

[15] Lundin P.M., Fu G.C. (2010). Asymmetric Suzuki cross-couplings of activated secondary alkyl electrophiles: Arylations of racemic alpha-chloroamides. J. Am. Chem. Soc..

[16] Oku N., Murakami M., Miura T. (2022). Photoassisted cross-coupling reaction of α-chlorocarbonyl compounds with arylboronic acids. Org. Lett..

[17] Rowe R.C., Sheskey P.J., Quinn M.E. (2009). Habdbook of Pharmaceutical Excipients.

[18] Ramos P. (2021). Compatibility studies of selected mucolytic drugs with excipients used in solid dosage forms: Thermogravimetry analysis. Farmacia.

[19] Ramos P. (2022). Thermal compatibility assessment of selected excipients used in the oral anti-cancer formulation containing busulfan. Farmacia.

[20] Kurek-Górecka A., Ramos P., Kłósek M., Bobela E., Czuba Z.P., Balwierz R., Olczyk P. (2023). Propolis as a cariostatic agent in lozenges and impact of storage conditions on the stability of propolis. Pharmaceutics.

[21] Pitucha M., Ramos P., Wojtunik-Kulesza K., Głogowska A., Stefańska J., Kowalczuk D., Drózd M., Augustynowicz-Kopeć E. (2023). Thermal analysis, antimicrobial and antioxidant studies of thiosemicarbazone derivatives. J. Therm. Anal. Calorim..

[22] Bartyzel A., Kaczor A.A., Głuchowska H., Pitucha M., Wróbel T.M., Matosiuk D. (2018). Thermal and spectroscopic studies of 2,3,5-trisubstituted and 1,2,3,5-tetrassubstituted indoles as non-competitive antagonists of GluK1/GluK2 receptors. J. Therm. Anal. Calorim..

[23] Saavedra-Leosa M.Z., Alvarez-Salasb C., EsneiderAlcala M.A., Toxqui-Terán A., Pérez-García S.A., RuizCabrera M.A. (2012). Towards an improved calorimetric methodology for glass transition temperature determination in amorphous sugars. CyTA—J. Food.

[24] Wei-Hsien H., Wen-Ting C., Ling-Chun C., HongLiang L., Shan-Yang L. (2018). Non-isothermal dehydration kinetics of glucose monohydrate, maltose monohydrate and trehalose dihydrate by thermal analysis and DSC-FTIR study. J. Biomed. Pharm. Sci..

[25] Zhao Z., Hayashi S., Xu W., Wu Z., Tanaka S., Sun S., Zhang M., Kanayama K., Umemura K. (2018). A Novel EcoFriendly Wood Adhesive Composed by Sucrose and Ammonium Dihydrogen Phosphate. Polymers.

[26] Wang C., Dou B., Song Y., Chen H., Yang M., Xu Y. (2014). Kinetic study on non-isothermal pyrolysis of sucrose biomass. Energy Fuels.

[27] Ilyes K., Casian T., Hales D., Borodi G., Rus L., Stiufius R., Tomuta I. (2021). Applying the principles of quality by design (QbD) coupled with multivariate data analysis (MVDA) in establishing the impact of raw material variability for extended release tablets. Farmacia.

[28] Lavor E.P., Navarro M.V.M., Freire F.D., Aragão C.F.S., Raffin F.N., Barbosa E.G., de Lima e Moura T.F.A. (2014). Application of thermal analysis to the study of antituberculosis drugs–excipient compatibility. J. Therm. Anal. Calorim..

[29] Listiohadi Y., Hourigan J.A., Sleigh R.W., Steele R.J. (2009). Thermal analysis of amorphous lactose and α-lactose monohydrate. Dairy. Sci. Technol..

[30] Suxia R., Xiuxuan S., Tingzhou L., Qinglin W. (2014). The effect of chemical and high-pressure homogenization treatment conditions on the morphology of cellulose nanoparticles. J. Nanomat..

[31] Kuthi F.A.A., Norzali N.R.A., Badri K.H. (2016). Thermal characteristics of microcrystalline cellulose from oil palm biomass. Malaysian J. Analyt. Sci..

[32] Katugampola P., Winstead C., Adeleke A. (2014). Thermal stability of carboxymethyl chitosan varying the degree of substitution. Int. J. Pharm. Sci. Invent..

[33] Liao S.K., Hung C.C., Lin M.F. (2004). A kinetic study of thermal degradations of chitosan/polycaprolactam blends. Macromolec. Res..

[34] Zhu J., Zhang S., Zhang B., Qiao D. (2017). Structural features and thermal property of propionylated starches with different amylose/amylopectin ratio. Int. J. Biol. Macromol..

[35] Kaczmarska K., Żymankowska-Kumon S., Grabowska B., Bobrowski A., Cukrowicz S. (2019). Study of thermal degradation of starch-based binder by TG-DTG-DSC Py-GC/MS and DRIFTS. Arch. Found. Engine..

[36] Tița B., Fuliaș A., Bandur G., Tiťa D., Babe V. (2011). Application of thermal analysis to study the compatibility of sodium diclofenac with different pharmaceutical excipients. Rev. Chim..

[37] Fuliaş A., Ledeţi I., Vlase G., Popoiu C., Hegheş A., Bilanin M., Vlase T., Gheorgheosu D., Craina M., Ardelean S. (2013). Thermal behaviour of procaine and benzocaine Part II: Compatibility study with some pharmaceutical excipients used in solid dosage forms. Chem. Cent. J..

[38] Wesołowski M., Rojek B. (2013). Thermogravimetric detection of incompatibilities between atenolol and excipients using multivariate techniques. J. Therm. Anal. Calorim..

[39] Rojek B., Wesołowski M. (2017). Compatibility studies of hydrocortisone with excipients using thermogravimetric analysis supported by multivariate statistical analysis. J. Therm. Anal. Calorim..

[40] Sip S., Paczkowska-Walendowska M., Rosiak N., Miklaszewski A., Grabańska-Martyńska K., Samarzewska K., Cielecka-Piontek J. (2021). Chitosan as Valuable Excipient for Oral and Topical Carvedilol Delivery Systems. Pharmaceuticals.

[41] Kepsutlu A.R., Savaser A., Ozkan Y., Dikmen N., Isimer A. (1999). Evaluation of chitosan used as an excipient in tablet formulations. Acta Pol. Pharm..

[42] Ray S.D. (2011). Potential aspects of chitosan as pharmaceutical excipient. Acta Pol. Pharm..

[43] Baldrick P. (2010). The safety of chitosan as a pharmaceutical excipient. Regul. Toxicol. Pharmacol..

[44] Echavarría A., Pagán J., Ibarz A. (2016). Kinetics of color development in glucose/amino acid model systems at different temperatures. Sci. Agropecu..

[45] Subert J., Cizmárik J. (2008). Application of instrumental colour measurement in development and quality control of drugs and pharmaceutical excipients. Die Pharm..

[46] MacDougall D.B., Mirjana Granov M. (2005). Relationship between Ultraviolet and Visible Spectra in Maillard Reactions and CIELAB Colour Space and Visual Appearance. Woodhead Publishing Series in Food Science, Technology and Nutrition.

[47] Sun Y., Lin L., Zhang P. (2021). Color Development Kinetics of Maillard Reactions. Ind. Eng. Chem. Res..

[48] Echavarría A.P., Pagán J., Ibarz A. (2013). Kinetics of color development of melanoidins formed from fructose/amino acid model systems. Food Sci. Tech. Intern..

[49] Arachchi S.J., Kim Y.J., Kim D.W., Oh S.C., Lee Y.B. (2017). Optimization of Maillard Reaction in Model System of Glucosamine and Cysteine Using Response Surface Methodology. Prev. Nutr. Food Sci..

[50] Sakiroff L.M., Chennell P., Yessaad M., Pereira B., Bouattour Y., Sautou V. (2022). Evaluation of color changes during stability studies using spectrophotometric chromaticity measurements versus visual examination. Sci. Rep..

[51] Golonka I., Wilk S., Musiał W. (2020). The influence of UV radiation on the degradation of pharmaceutical formulations containing quercetin. Molecules.

[52] Akhtar F., Gul S., Ashfaq S., Rehman I., Mirza A.Z. (2020). UV spectroscopic method for optimization and determination of glibenclamide in bulk, pharmaceutical dosage form and its application for in vitro interaction studies. J. Anal. Test..

[53] Kukulski T., Wacławek S., Silvestri D., Krawczyk K., Padil V.V.T., Fryczkowski R., Janicki J., Černík M. (2020). A Polymeric Composite Material (rGO/PANI) for Acid Blue 129 Adsorption. Polymers.

[54] Zhang H., Ou J., Yao Y., Wang H., Liu Z., Wei Y., Ye M. (2017). Facile Preparation of Titanium(IV)-Immobilized Hierarchically Porous Hybrid Monoliths. Anal. Chem..

[55] Silvestri D., Wacławek S., Venkateshaiah A., Krawczyk K., Sobel B., Padil V.V.T., Černík M., Varma R.S. (2020). Synthesis of Ag Nanoparticles by a Chitosan-Poly(3-Hydroxybutyrate) Polymer Conjugate and Their Superb Catalytic Activity. Carbohydr. Polym..

[56] Silvestri D., Wacławek S.K., Ramakrishnan R., Venkateshaiah A., Krawczyk K., Padil V.V.T., Sobel B., Černík M. (2019). The Use of a Biopolymer Conjugate for an Eco-Friendly One-Pot Synthesis of Palladium-Platinum Alloys. Polymers.

[57] ICH (2003). ICH Harmonised Tripartite Guideline: Stability Testing of New Drug Substances and Products, Q1A (R2).

[58] Ding P., Ling Y.S. (2014). Brownikg assessment methods and polyphenol oxidase in UV-C irradiated Berangan banana fruit. Intern. Food Res. J..

