# Supplementary material for: Thermal Compatibility of New ACEI Derivatives with Popular Excipients Used to Produce Solid Pharmaceutical Formulations
Source: Pharmaceuticals (Basel). 2024 Oct 3;17(10):1323. doi: 10.3390/ph17101323 (PMC11510466; doi:10.3390/ph17101323)
Supplement: Supplementary file 1 [file pharmaceuticals-17-01323-s001.zip › pharmaceuticals-3203487-supplementary.pdf]

Supplementary material

Thermal compatibility of new ACEI derivatives with popular excipients used to produce solid pharmaceutical formulations

Mateusz Broncel [1,2]*, Anna Juszczak [3], Wojciech Szczolko [4], Daniele Silvestri [5], Agnieszka Białek-Dratwa [6], Stanisław Wacławek [5], Oskar Kowalski [6] and Paweł Ramos [1]*

[1] Department of Biophysics, Faculty of Pharmaceutical Sciences in Sosnowiec, Medical University of Silesia, Katowice, Jedności 8, 41-200 Sosnowiec, Poland; mateusz_broncel@interia.pl (M.B.), pawelramos@sum.edu.pl (P.R.)
[2] Doctoral School, Medical University of Silesia, Katowice, Jedności 8, 41-200 Sosnowiec, Poland; mateusz_broncel@interia.pl (M.B.)
[3] Chair and Department of Pharmaceutical Chemistry, Poznan University of Medical Sciences, Rokietnicka 3, 60-806 Poznan, Poland; anna.juszczak28@gmail.com (A.J.)
[4] Chair and Department of Chemical Technology of Drug, Poznan University of Medical Sciences, Rokietnicka 3, 60-806 Poznan, Poland; wszczolko@ump.edu.pl (W.S.)
[5] Institute for Nanomaterials, Advanced Technologies and Innovation, Technical University of Liberec, Stdentská 2, 460 01 Liberec, Czech Republic; daniele.silvestri@tul.cz (D.S.), stanislaw.waclawek@tul.cz (S.W.)
[6] Department of Human Nutrition, Department of Dietetics, Faculty of Public Health in Bytom, Medical University of Silesia in Katowice, Jordana 19, 41-808 Zabrze, Poland; abialek@sum.edu.pl (A.B.D.), okowalski@sum.edu.pl (O.K.)

* Correspondence: Mateusz.broncel@interia.pl; Tel.: +48-519-623-700, pawelramos@sum.edu.pl; Tel.: +48-792-280-882

General procedures……………………………………………………………...……………2
NMR data…….…...…………….………………………………………………….....….…..3
HRMS spectra of IND-1 and IND-2…………………………………………………………4
MS spectra of 2………………………………………………………………………………..6
UV-Vis spectra of IND-1 and IND-2………………………………………………………...7
Table S1. The table presents commercial pharmaceutical preparations containing individual ACE inhibitors and the tested excipients……………………………………….8

General procedures

All reactions were conducted in oven-dried glassware under argon. All solvents were rotary evaporated at or below 50ºC. Reaction temperatures reported refer to external bath temperatures. Solvents and all reagents were obtained from commercial suppliers and used without further purification. Melting points were obtained on a "Stuart" Bibby apparatus and are uncorrected. Dry flash column chromatography was carried out on Merck silica gel 60, particle size 40-63 μm. Thin layer chromatography (TLC) was performed on silica gel 60A F_{254} plates and visualized with UV (λ_{max} 254 or 365 nm). UV-Vis spectra were recorded on a Hitachi UV-Vis U-1900; λ_{max} (logε), nm. Mass spectra (ES) were carried out by the Wielkopolska Centre for Advanced Technologies in Poznan. ^{1}H NMR, ^{13}C NMR spectra were recorded using Agilent DD2800 spectrometer at the NanoBioMedical Centre in Poznan, or Bruker Avance III 700 and Avance III 500 spectrometers at the Institute of Bioorganic Chemistry PAS. Chemical shifts (δ) are quoted in parts per million (ppm) and are referred to as a residual solvent peak. Coupling constants (*J*) are quoted in Hertz (Hz). The abbreviations s, t and m refer to singlet, triplet and multiplet, respectively.

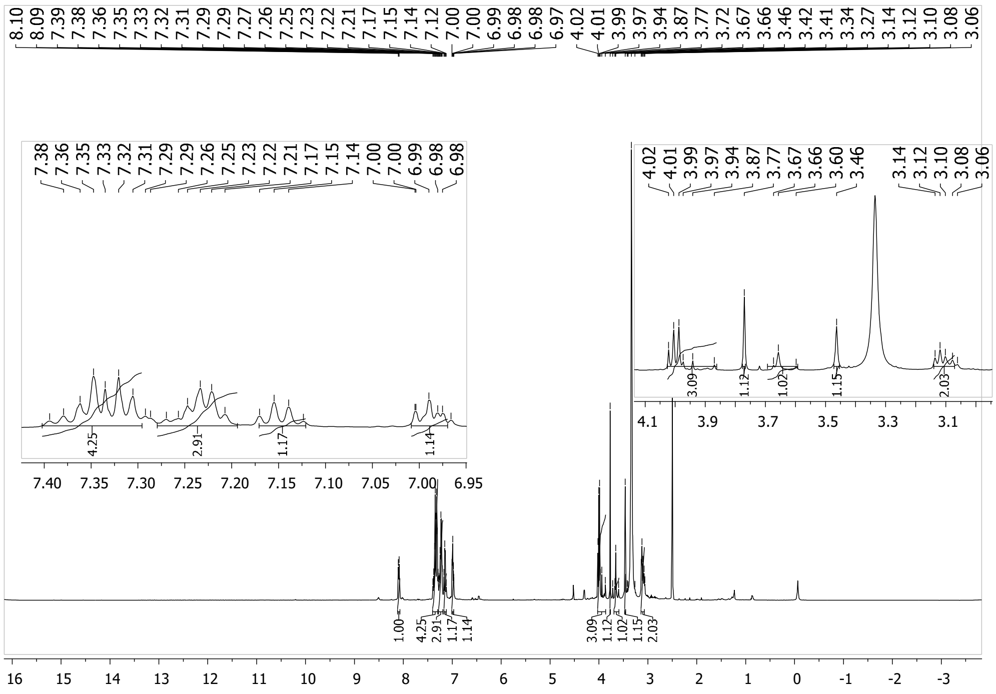

^{1}H NMR of 2-(benzylamino)-1-(2,3-dihydroindol-1-yl)ethanone in DMSO-d_6

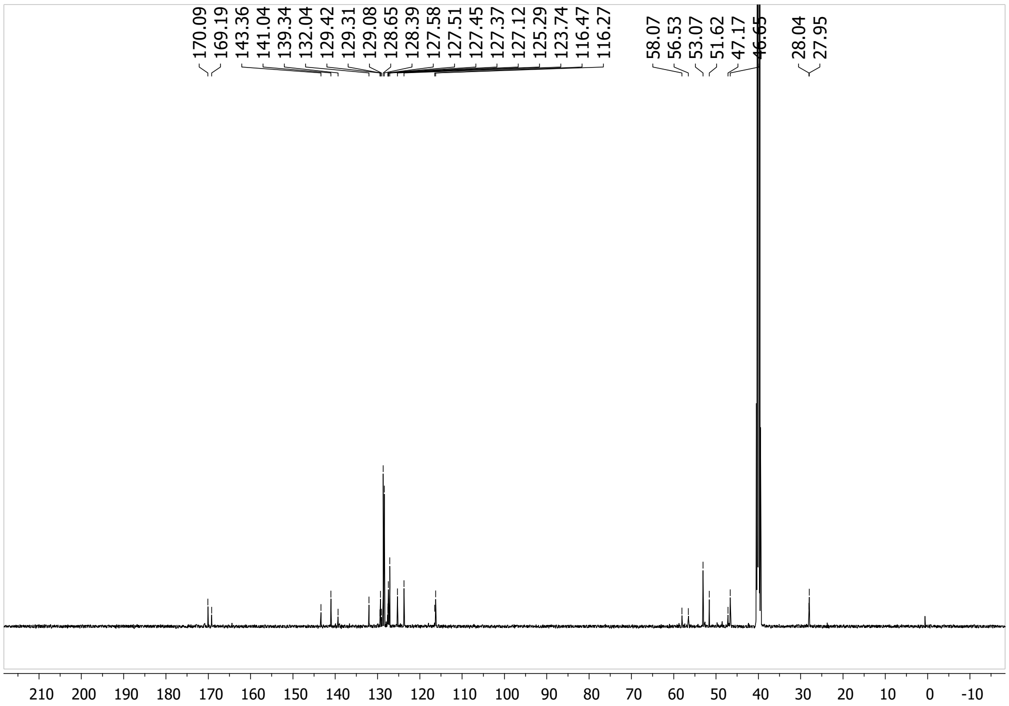

^{13}C NMR of 2-(benzylamino)-1-(2,3-dihydroindol-1-yl)ethanone in DMSO-d_6

Display Report

Acquisition Parameter

Source Type	ESI	Ion Polarity	Positive	Set Nebulizer	0.3 Bar
Focus	Active	Set Capillary	4500 V	Set Dry Heater	200 °C
Scan Begin	50 m/z	Set End Plate Offset	-500 V	Set Dry Gas	4.0 l/min
Scan End	400 m/z	Set Charging Voltage	2000 V	Set Divert Valve	Source
		Set Corona	0 nA	Set APCI Heater	0 °C

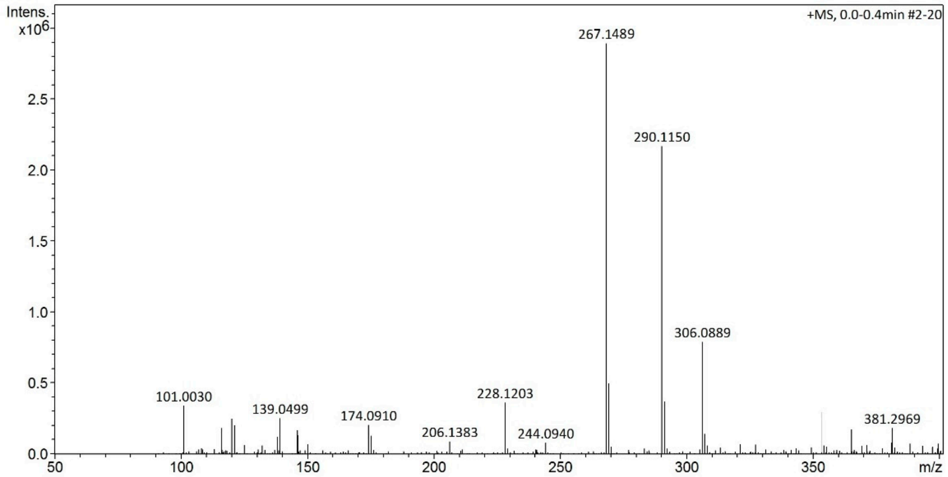

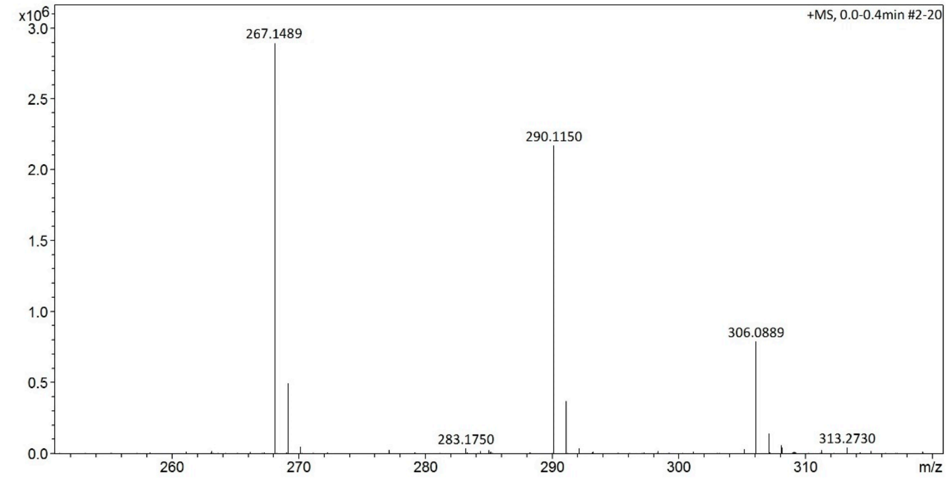

HRMS spectra of **IND-1**

Display Report

Acquisition Parameter

Source Type	ESI	Ion Polarity	Positive	Set Nebulizer	0.3 Bar
Focus	Active	Set Capillary	4500 V	Set Dry Heater	200 °C
Scan Begin	50 m/z	Set End Plate Offset	-500 V	Set Dry Gas	4.0 l/min
Scan End	400 m/z	Set Charging Voltage	2000 V	Set Divert Valve	Source
		Set Corona	0 nA	Set APCI Heater	0 °C

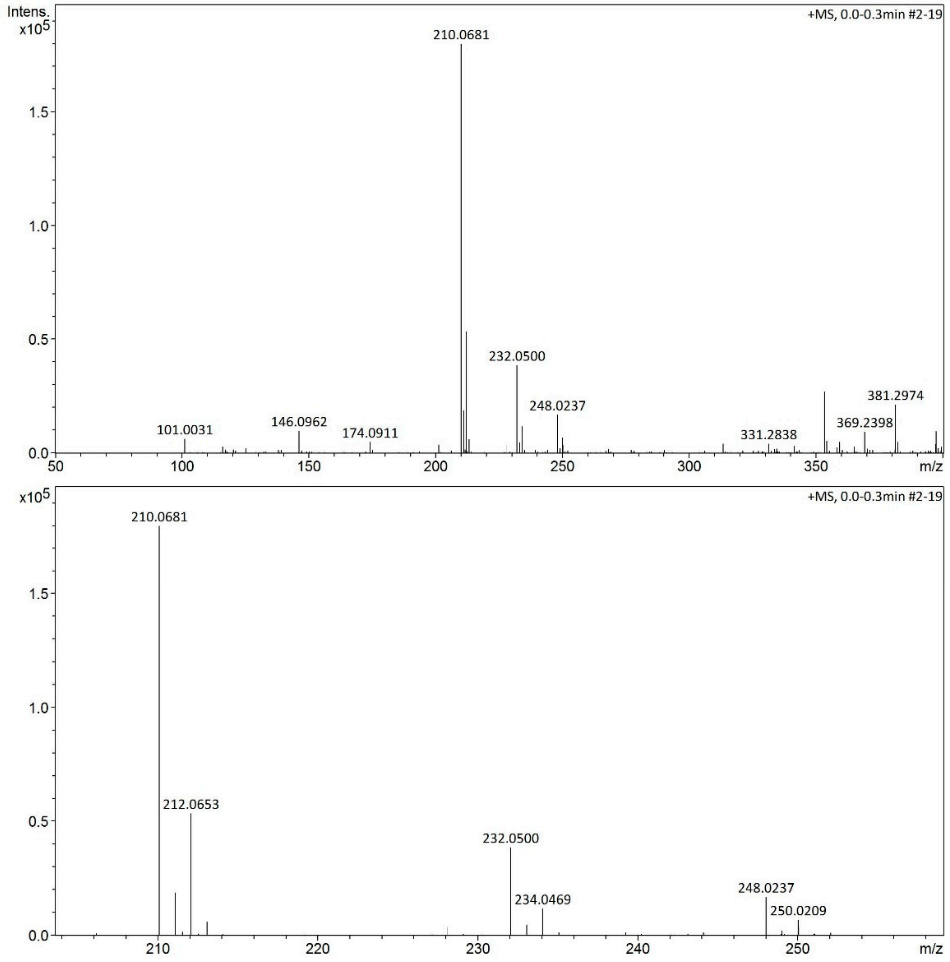

HRMS spectra of **IND-2**

Spectrum Name: AJ_41
Start Ion: 10
End Ion: 1200
Source: ESI - 2.5kV 350C
Capillary: 150V 300C Offset: 25V Span: 0V

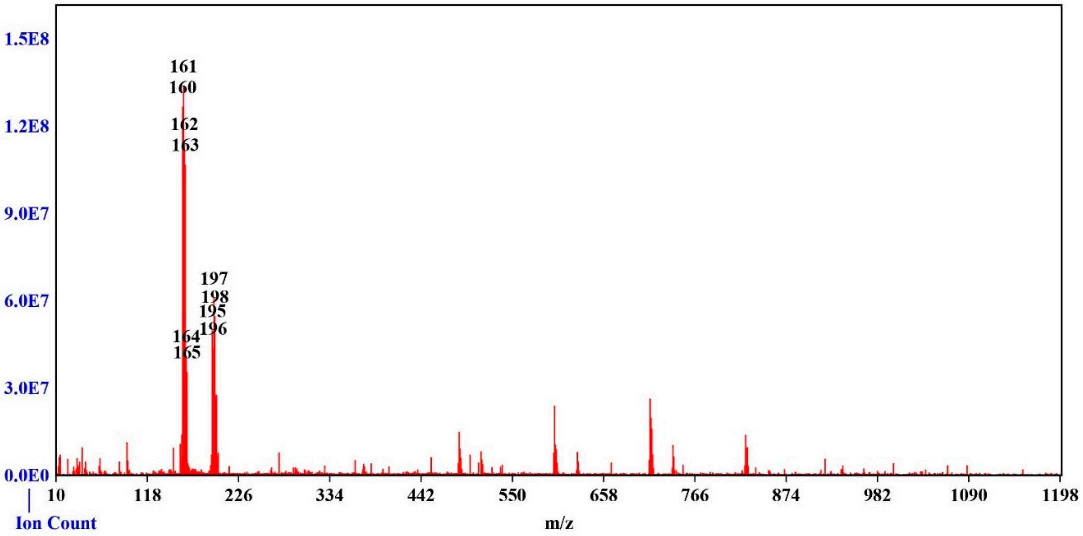

MS spectra of **2**

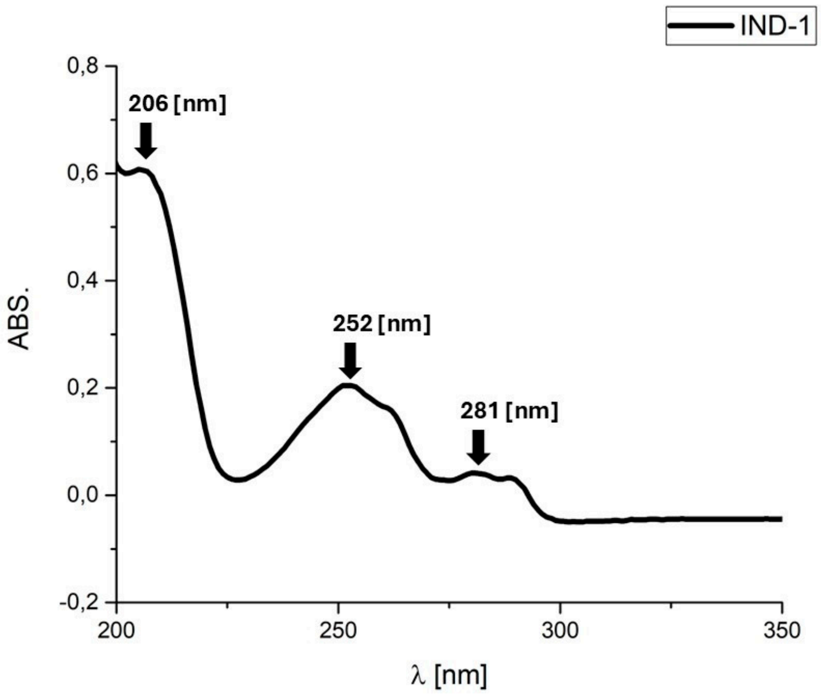

UV-Vis spectra of IND-1

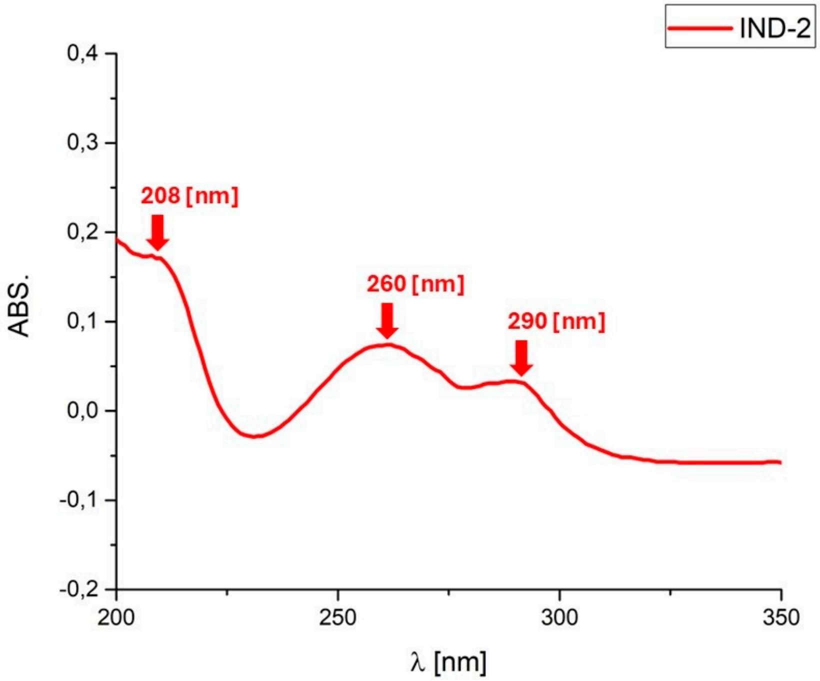

UV-Vis spectra of IND-2

Table S1. The table presents commercial pharmaceutical preparations containing individual ACE inhibitors and the tested excipients.

API	Lactose monohydrate	Microcrystalline cellulose	Starch from corn	Magnesium stearate
**Ramipril**	**Polpril** (2.5mg, 5mg, 10mg); **Vivace** (2.5mg, 5mg, 10mg); **Axtil** (2.5mg, 5mg, 10mg)	**Piramil** (1.25mg, 2.5mg, 5mg, 10mg); **Ramicor** (2.5mg, 5mg, 10mg); **Tritace** (2.5mg, 5mg, 10mg);	**Polpril** (2.5mg, 5mg, 10mg); **Vivace** (2.5mg, 5mg, 10mg); **Axtil** (2.5mg, 5mg, 10mg); **Piramil** (1.25mg, 2.5mg, 5mg, 10mg); **Ramicor** (2.5mg, 5mg, 10mg); **Tritace** (2.5mg, 5mg, 10mg);	
**Perindopril**	**Prestarium** (2.5mg, 5mg); **Perindopril Teva** (5mg, 10mg); **Prenessa** (4mg, 8mg); **Pricoron** (5mg, 10mg); **Vidotin** (4mg, 8mg);	**Prenessa** (4mg, 8mg); **Vidotin** (4mg, 8mg);	**Perindopril Teva** (5mg, 10mg); **Pricoron** (5mg, 10mg);	**Prestarium** (2.5mg, 5mg); **Perindopril Teva** (5mg, 10mg); **Prenessa** (4mg, 8mg); **Pricoron** (5mg, 10mg); **Vidotin** (4mg, 8mg);
**Enalapril**	**Benalapril** (5mg, 10mg, 20mg); **Enarenal** (5mg, 10mg, 20mg)		**Enarenal** (5mg, 10mg, 20mg)	**Benalapril** (5mg, 10mg, 20mg); **Enarenal** (5mg, 10mg, 20mg)
**Captopril**	**Captopril Jelfa** (12.5mg, 25mg, 50mg); **Captopril Polfarmex** (12.5mg, 25mg, 50mg)	**Captopril Jelfa** (12.5mg, 25mg, 50mg); **Captopril Polfarmex** (12.5mg, 25mg, 50mg)		**Captopril Polfarmex** (12.5mg, 25mg, 50mg)
**Benazepril**	**Lotensin** (20mg)	**Lotensin** (20mg)	**Lotensin** (20mg)	
**Quinapril**	**Acurenal** (5mg, 10mg, 20mg); **Pulsaren** (10mg, 20mg);			**Acurenal** (5mg, 10mg, 20mg); **Pulsaren** (10mg, 20mg)

**Cilazapril**	**Cazaprol** (1mg, 2.5mg, 5mg); **Cilan** (0.5mg, 1mg, 2.5mg, 5mg); **Inhibace** (1mg, 2.5mg, 5mg); **Symibace** (2.5mg)		**Cazaprol** (1mg, 2.5mg, 5mg); **Cilan** (0.5mg, 1mg, 2.5mg, 5mg); **Inhibace** (1mg, 2.5mg, 5mg); **Symibace** (2.5mg)	
**Fosinopril**		Zofenil (7.5mg, 30mg)	Zofenil (7.5mg, 30mg)	
**Lisinopril**			**LisiHEXAL** (5mg, 10mg, 20mg); **Lisinopril Aurivitas** (10mg, 20mg); **Lisinoratio** (5mg, 10mg, 20mg); **Lisiprol** (5mg, 10mg, 20mg); **Ranopril** (5mg, 10mg, 20mg)	**LisiHEXAL** (5mg, 10mg, 20mg); **Lisinopril Aurivitas** (10mg, 20mg); **Lisinoratio** (5mg, 10mg, 20mg); **Lisiprol** (5mg, 10mg, 20mg); **Ranopril** (5mg, 10mg, 20mg)
**Imidapril**	**Tanatril** (10mg)		**Tanatril** (10mg)	
**Trandolapril**	**Gopten** (0.5mg, 2mg, 4mg)		**Gopten** (0.5mg, 2mg, 4mg)	
